# Post-traumatic stress disorder during the Covid-19 pandemic: a national, population-representative, longitudinal study of U.S. adults

**DOI:** 10.1038/s44184-024-00059-w

**Published:** 2024-04-10

**Authors:** Salma M. Abdalla, Catherine K. Ettman, Samuel B. Rosenberg, Ruochen Wang, Gregory H. Cohen, Sandro Galea

**Affiliations:** 1 Department of Epidemiology, Boston University School of Public Health, Boston, MA USA; 2 Johns Hopkins Bloomberg School of Public Health, Baltimore, MD USA

**Keywords:** Psychology, Human behaviour, Psychiatric disorders

## Abstract

Substantial literature documents the impact of mass traumatic events on post-traumatic stress disorder (PTSD) in populations. However, the trajectory of PTSD in the US population during the pandemic and the association between assets, Covid-19 related stressors, and PTSD over time remains unclear. The Covid-19 and Life Stressors Impact on Mental Health and Well-Being (CLIMB) is a nationally representative, longitudinal panel of US adults in Spring 2020 (*N* = 1270), 2021 (*N* = 1182), and 2022 (*N* = 1091). Using the four-item PC-PTSD-4, we assessed the prevalence of probable PTSD in the US population over three years. Using generalized estimating equations (GEE) and logistic regression at each wave, we estimated associations of demographics, assets, and stressors with probable PTSD. Here we report that the overall prevalence of PTSD decreases from 22.2% in 2020 to 16.8% in 2022 (*p* = 0.02). Persons with household incomes below $20,000 report higher prevalence of probable PTSD compared to other income groups. The GEE model shows higher odds of probable PTSD among persons with household incomes below $20,000 (OR = 2.17 (95%CI: 1.35,3.50)) relative to $75,000 or more; and high stressor scores (OR = 2.33 (95%CI: 1.72,3.15)) compared to low stressor scores. High stressor scores are associated with higher odds of probable PTSD in 2020 (OR = 2.69 (95%CI: 1.56,4.66)), 2021 (OR = 4.58 (95%CI: 2.52,8.30)), and 2022 (OR = 3.89 (95%CI: 2.05,7.38)) compared to low stressor scores. This analysis highlights the pandemic’s prolonged influence on population mental health, particularly among persons with fewer economic assets and those experiencing more pandemic-related stressors. Reducing mental health disparities requires interventions to address inequities.

## Introduction

The Covid-19 pandemic, similar to other mass traumatic events, has been associated with a substantial increase in adverse mental health indicators. Several studies have documented worsening mental health in the United States and globally, including a rise in post-traumatic stress disorder (PTSD)^1–3^. This rise in PTSD can be attributed to the pandemic’s role as a mass traumatic event^3–6^. This is consistent with literature from other mass traumatic events such as natural disasters and other disease outbreaks^5,7–12^.

Previous work has illustrated the role social and economic stressors play in the rise of adverse mental health outcomes during the pandemic, including PTSD^3,6,13–15^. This is consistent with research following other mass traumatic events. Experiencing event-related stressors—such as deaths of loved ones or friends, displacement, property damage, and financial problems—has been associated with greater PTSD risk^9,10,16–18^. However, evidence of the relation between experiencing specific pandemic-related stressors and PTSD remains limited in the literature.

There is ample research on the long term mental health consequences of experiencing mass traumatic events, including PTSD, well beyond the initial event^4,5,19–23^. Studies assessing PTSD following the Covid-19 pandemic have generally been cross-sectional or have restricted the longitudinal observation period to under a year^24^. Relatively few longitudinal studies assessed PTSD one year or longer after the start of the pandemic or assessed the long term association between exposure to pandemic-related stressors and PTSD^25–28^.

We aim to address this gap in the literature by documenting the course of probable PTSD following the pandemic and the associations between exposure to social and economic stressors and PTSD over time using a nationally representative longitudinal cohort. Specifically, this paper aims to (1) estimate probable PTSD prevalence three years into the Covid-19 pandemic; (2) evaluate the association between experiencing social and economic stressors and PTSD; and (3) assess how assets relate to probable PTSD throughout the pandemic.

## Methods

### Study population and Sample size

Using a random sample of adult participants in the AmeriSpeak Panel, we collected nationally representative data for the Covid-19 and Life Stressors Impact on Mental Health and Well-Being Study (CLIMB). Additional details on the recruiting process are available in a previous publication^6^. Online and telephone surveys occurred near the beginning of the Covid-19 pandemic from March 31, 2020 to April 13, 2020 (Time 1, T1), one year into the Covid-19 pandemic from March 23, 2021 to April 19, 2021 (Time 2, T2), and two years into the Covid-19 pandemic from March 22, 2022 to April 18, 2022 (Time 3, T3).

We calculated two post-stratification weights: one for participants who completed at least two waves and one for each wave. The participation frequency and completion rate are available in a previous publication^6^. The institutional review boards of the National Opinion Research Center (NORC) at Chicago University and Boston University Medical Campus (H-39986) approved the study. NORC obtained written consent from study participants when they first enrolled in the AmeriSpeak Panel.

To maximize sample size and accuracy of estimations, we used complete samples for participants who responded to T1 (*N* = 1270), T2 (*N* = 1182), or T3 (*N* = 1091). For the cross-sectional analyses, we removed participants from T1 who did not complete at least two waves (*N* = 199) or were missing a PTSD score from T1 (*N* = 13), T2 (*N* = 15), or T3 (*N* = 12). Participants with only one remaining PTSD score were also removed from T1 (*N* = 9) and T2 (*N* = 3).

### Demographic characteristics and other key variables

Gender was defined as a binary variable: man or woman. Age was defined as a categorical variable: 18–39, 40–59, or 60 years or older. Ethnicity was collapsed into a mutually exclusive categorical variable: non-Hispanic Asian, non-Hispanic Black, Hispanic, Multiple or other, or non-Hispanic white, ascertained from participant self-reporting. Educational attainment was defined as a binary variable: less than a college degree or college degree or more.

We measured assets and household size. In this analysis, assets refer to household income, household savings, debts, and home ownership. Household income was defined as a categorical variable: $19,999 or less; $20,000–$44,999; $45,000–$74,999; and $75,000 or more. Household savings was defined as a binary variable: $19,999 or less or $20,000 or greater. Home ownership was defined as a categorical variable: Own, Rent, or Other. Debt was defined as a categorical variable, consisting of all household debts (college loans, mortgage, etc.): no debt; $9999 or less; and $10,000 or more. Household size was a continuous variable that included all persons living in the home, ranging from one to seven or more.

History of Covid-19 infection was defined in response to the question at T1 and T2, “Has a doctor or other health professional ever told you that you had coronavirus or COVID-19?” and the question at T3, “Have you ever tested positive for coronavirus or COVID-19?” Covid-19 vaccine status was measured at T2 and T3 by asking participants, “Have you received at least one shot of the COVID-19 vaccine?”

### Pandemic related stressors

Participants were asked if they had ever experienced a series of enumerated stressors due to Covid-19 at T1 and if they had experienced Covid-19 induced stressors in the past 12 months at T2 and T3. We measured 15 stressors based on previous studies conducted after traumatic events and as previously published^3,13^. Stressor counts were divided into three categories based on terciles at T1 and T2: low stressor count (zero to three stressors), medium stressor count (four to five stressors), and high stressor count (six or more stressors). The T2 and T3 survey waves had additional stressors that were not in T1, which we used for developing Figs. 4A, B and 5. The additional stressors we based on our evolving knowledge of the consequences of the pandemic. These stressors included: being forced to leave campus, experiencing a housing eviction or losing housing, not having enough food to eat, losing health insurance, and experiencing divorce or separation.

### Post-traumatic stress disorder symptoms

Participants completed the four-item PTSD checklist (PC-PTSD-4) to assess the presence of probable PTSD. Probable PTSD was defined as a score of 3 or greater^29^. We also analyzed each PTSD symptom separately for each measured stressor at T2 and T3. While we did not assess past traumatic events, we anchored the survey in the context of the Covid-19 pandemic as a mass traumatic event. For example, we prefaced the PC-PTSD-4 questions with the following format: “Has the coronavirus or COVID-19 outbreak been so frightening, horrible, or upsetting that you had nightmares about it or thought about it when you did not want to?”

### Statistical analysis

To adjust for sample selection factors, we weighted all analyses using multiple post-stratification survey weights: one at each time point and one for participants who completed at least two time points. We used either the weights for one time point or the weights for at least two time points depending on the type of analysis. First, we summed stressors and created terciles of low, medium, and high stressor sums. Second, we stratified probable PTSD prevalence by demographic characteristics at T1, T2, and T3, comparing the prevalence using a two-tailed chi-square test and two-sample t-test. Third, to understand how economic circumstances at the beginning of the pandemic shaped the course of PTSD, we anchored household income and household savings to T1 and estimated probable PTSD prevalence at T1, T2, and T3 by household income and household savings at T1.

Fourth, we calculated the overall prevalence of probable PTSD for the sum of all stressors and by each stressor at T2 and T3. Fifth, we estimated the adjusted odds of probable PTSD across time using general estimating equations (GEE) to account for repeated measures, controlling for gender, age, ethnicity, educational attainment, income, savings, home ownership, household size, history of Covid-19 infection, Covid-19 vaccine status (T2 and T3 only), and stressor category. Sixth, using logistic regression, we estimated the adjusted odds of probable PTSD at T1, T2, and T3 in each time-specific sample.

We conducted analyses in R v4.2.1, complementing the process with the packages data.table, here, survey, haven, gt, gtsummary, tidyverse, geepack, geeasy, geeM, srvyr, convey, paletteer, ggthemes, pacman, and MESS^30–46^.

## Results

### Sample demographics

Participants in the cross-sectional samples numbered 1252 in T1, 1165 in T2, and 1081 in T3, consisting of responders who had a PTSD score for at least two waves. Although we presented the weighted sample of responders at T1 in a separate paper^3^, these criteria restricted the sample, resulting in different demographic distributions. In the weighted sample of responders at T1 (*N* = 1252), over half were women (51.0%), 35.2% were non-white, 35.7% had a college degree or more, 69.1% had a household income below $75,000, 58.0% had less than $20,000 in household savings, and 40.9% had $10,000 or more in household debt. In the weighted sample of responders at T2 (*N* = 1165), over half were women (51.7%), 37.2% were non-white, a third had a college degree or more (35.2%), 69.4% had a household income below $75,000, over half had less than $20,000 in household savings (52.8%), 42.4% had $10,000 or more in household debt, 11.1% had had a previous Covid-19 infection, and less than half had had a Covid-19 vaccine (42.9%). In the weighted sample of responders at T3 (*N* = 1081), over half were women (52.1%), 37.0% were non-white, a third had a college degree or more (34.6%), two-thirds had a household income below $75,000 (66.0%), over half had less than $20,000 in household savings (52.4%), 37.3% had $10,000 or more in household debt, a third had had a Covid-19 infection (33.2%), and 81.1% had had a Covid-19 vaccine. The longitudinal analytic sample included only participants who had at least one response to all variables included in the model, with the final sample consisting of 1159 participants from T1, 1070 from T2, and 954 from T3.

Responders at T2 (*N* = 1165) and non-responders at T2 (*N* = 93) were demographically comparable at T1, except for ethnicity and household savings. Compared to responders at T2, more non-responders were non-white, and more non-responders had under $20,000 in household savings. (Supplementary Table 1). Responders at T3 (*N* = 1081) and non-responders at T3 (*N* = 177) were demographically comparable at T1, except for age, ethnicity, debt, and household size. More non-responders were 18–39 years, more non-responders were non-white, and non-responders had larger household sizes compared to responders at T3 (Supplementary Table 2).

### Probable PTSD prevalence over time

Table 1 shows the frequency and weighted percentage of the sample population with probable PTSD at T1, T2, and T3. Supplementary Table 3 provides the distribution of PTSD scores by wave. Probable PTSD prevalence decreased in each subsequent year compared to the previous year. The difference between probable PTSD prevalence at T1 and T3 was statistically significant. A higher prevalence of probable PTSD occurred in women compared to men, with statistical significance at T1 and T2. The youngest age group had the highest prevalence of probable PTSD; this prevalence decreased with older age, with statistical significance at T1. Across each wave, Hispanic participants had the highest prevalence of probable PTSD, followed by white participants at T1 and by Black participants at T2, and by multiple or other ethnicity participants at T3.

**Table 1:** Associations of demographic characteristics, assets, and stressors with probable PTSD in 2020, 2021, and 2022

	2020 (T1)			2021 (T2)			2022 (T3)		
	No.	%	*p* value	No.	%	*p* value	No.	%	*p* value
Characteristics									
Total	261	22.2	0.6780 (T1:T2)	211	21.2	0.07(T2:T3)	154	16.8	<0.02 (T1:T3)
Gender			0.01			0.03			0.2
Men	99	18.2		82	17.2		60	14.8	
Women	162	25.9		129	24.9		94	18.6	
Age			<0.01			0.41			0.18
18–39	121	26.5		84	22.0		59	19.2	
40–59	98	25.6		79	23.3		55	18.9	
60+	42	13.3		48	17.8		40	12.7	
Ethnicity			0.19			0.54			0.91
Asian, non-Hispanic	3	11.0		3	11.4		4	17.0	
Black, non-Hispanic	24	21.6		18	22.9		18	18.2	
Hispanic	56	29.1		44	26.4		28	19.5	
Multiple or other	9	13.4		12	18.9		8	18.8	
White, non-Hispanic	169	21.7		134	20.2		96	15.7	
Educational attainment			0.17			0.73			0.07
Less than a college degree	184	23.7		137	21.6		105	18.7	
College degree or more	77	19.4		74	20.4		49	13.3	
Income ($)			0.01			<0.01			<0.01
≤19,999	59	33.5		44	25.3		27	30.3	
20,000–44,999	69	20.4		62	27.8		45	18.6	
45,000–74,999	59	20.4		56	22.0		33	16.6	
≥75,000	70	19.0		45	12.4		47	11.3	
Savings ($)			<0.01			0.01			<0.01
≤19,999	180	26.6		136	25.7		104	23.5	
≥20,000	77	16.5		70	16.4		50	11.3	
Debt			<0.01			<0.01			0.10
No debt	26	13.6		38	15.5		20	10.7	
≤9999	99	23.1		62	16.9		63	20.3	
≥10,000	132	25.2		108	27.9		71	19.0	
Home ownership			0.37			0.06			<0.01
Own	141	20.9		113	19.8		77	13.1	
Rent	109	23.8		87	21.0		63	20.1	
Other	11	31.2		11	40.6		14	44.4	
Household size (mean)	3.4	3.4	0.04	3.2	3.2	0.98	2.4	2.4	0.49
Covid-19 infection			0.05			0.17			0.11
Did not have Covid-19 infection	258	21.9		182	20.2		97	14.9	
Had Covid-19 infection	3	57.3		29	29.5		57	20.6	
Covid-19 vaccine			NA			0.49			0.02
Did not have Covid-19 vaccine	NA	NA		113	20.1		22	10.5	
Had Covid-19 vaccine	NA	NA		98	22.6		130	18.3	
Stressor Category			<0.01			<0.01			<0.01
Low (0–3)	38	13.9		33	11.2		39	9.7	
Medium (4–5)	73	18.6		48	16.6		44	17.1	
High (6+)	150	30.7		127	35.6		65	33.5	

Frequencies unweighted; percentages weighted. T1 weights used for T1 estimates; T2 weights used for T2 estimates; T3 weights used for T3 estimates. One-sample t-tests used to calculate p-values between waves. Chi-square tests of independence used to calculate *p* values.

Probable PTSD defined by Primary Care PTSD Screen for DSM-5 (PC-PTSD-4) score of 3 or greater.

Data source: COVID-19 and Life Stressors Impact on Mental Health and Well-being study. Time 1 collected from March 31, 2020, to April 13, 2020. Time 2 collected from March 23, 2021, to April 19, 2021. Time 3 collected from March 22, 2022, to April 18, 2022. Time 1, *N* = 1252; Time 2, *N* = 1165; Time 3, *N* = 1081.

Covariates collected at Time 1 were used for Time 1 estimates; covariates collected at Time 2 were used for Time 2 estimates. Covariates collected at Time 3 were used for Time 3 estimate.

At T1, 26 participants were missing household income, 32 participants were missing household savings, one participant was missing data for COVID-19 infection, and nine participants were missing stressors. At T2, 38 participants were missing household income, 47 participants were missing household savings, and 43 participants were missing stressors. At T3, 32 participants were missing household income, 46 participants were missing household savings, one participant was missing data for COVID-19 infection, 11 participants were missing data for COVID-19 vaccination, and 79 participants were missing stressors.

Participants with less than a college degree had a higher prevalence of probable PTSD than participants with a college degree or more. Probable PTSD prevalence was higher in participants with a history of Covid-19 infection. Nearly a quarter of participants who had received at least one Covid-19 vaccine at T2 had probable PTSD, compared to 20.1% of participants who had not received the Covid-19 vaccine. At T3, having the Covid-19 vaccine was associated with a higher prevalence of probable PTSD, reaching statistical significance (Table 1).

### Assets and probable PTSD prevalence

**Fig. 1:**
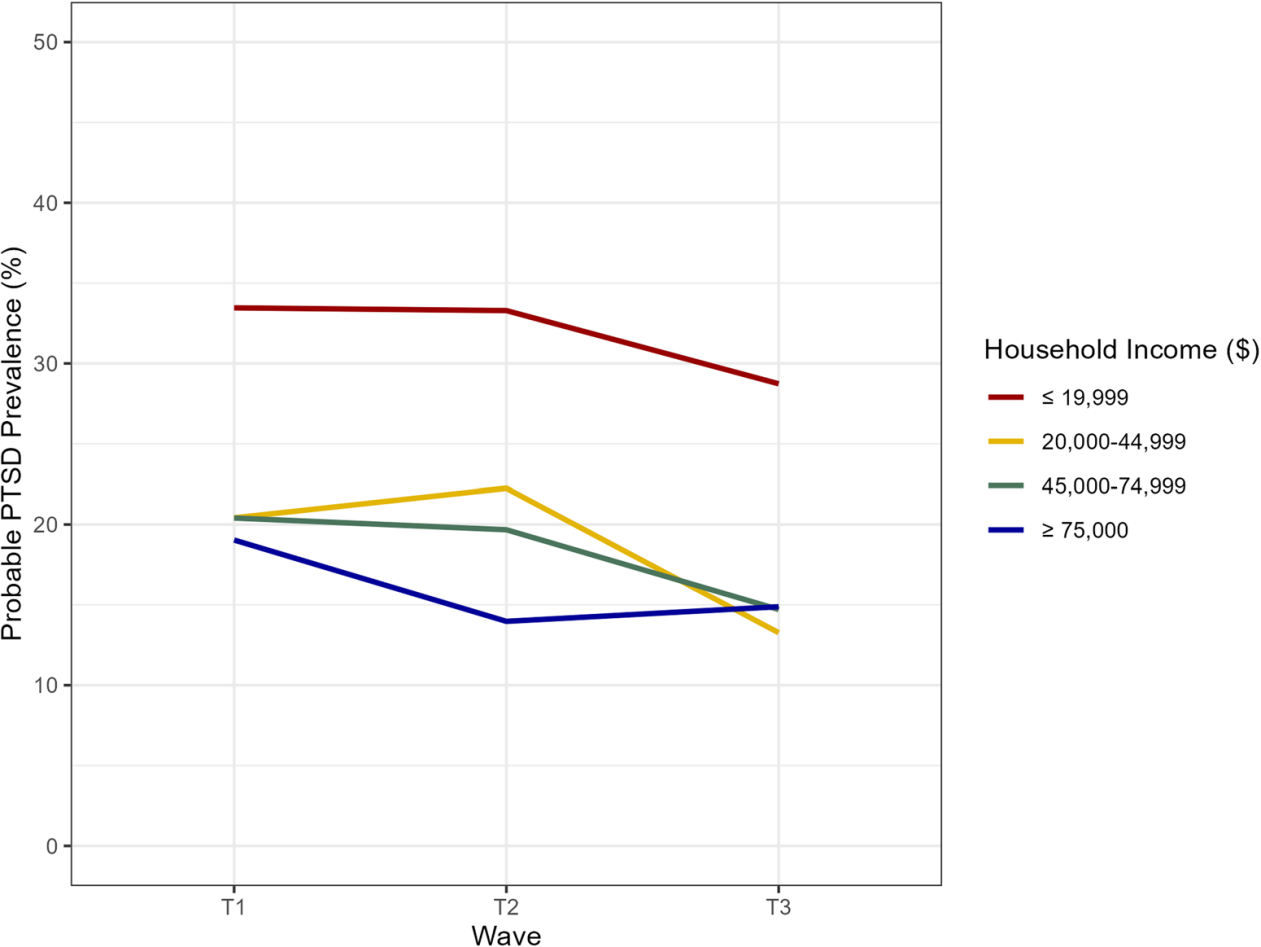
Prevalence of probable PTSD by income level and data collection wave, anchored income to T1. Household income data anchored to T1. Respondents have same household income in T2 and T3 as they did in T1. Household income in $USD. Data weighted. T1 weights used for T1 estimates; T2 weights used for T2 estimates; T3 weights used for T3 estimates Probable PTSD defined by Primary Care PTSD Screen for DSM-5 (PC-PTSD-4) score of 3 or greater. Data source: COVID-19 and Life Stressors Impact on Mental Health and Well-being study. Time 1 collected from March 31, 2020, to April 13, 2020. Time 2 collected from March 23, 2021, to April 19, 2021. Time 3 collected from March 22, 2022, to April 18, 2022. At T1, 26 respondents had missing or unknown household income. At T2, 31 respondents had missing or unknown household income. At T3, 27 respondents had missing or unknown household income.

The group with the lowest household income (≤$19,999) had the highest prevalence at T1 and T3. Prevalence of probable PTSD generally decreased with increasing income, except for T2, when the $20,000–$44,999 group had the highest prevalence of probable PTSD. There was a statistically significant association between household income and probable PTSD. Figure 1 shows the probable PTSD prevalence at each time point, stratified by household income level anchored to T1. Participants in the lowest household income group at T1 had the highest prevalence of probable PTSD at any time point, and each increasing income level had a lower prevalence of probable PTSD at T1 and T2. Supplementary Fig. 1 includes error bars.

**Fig. 2:**
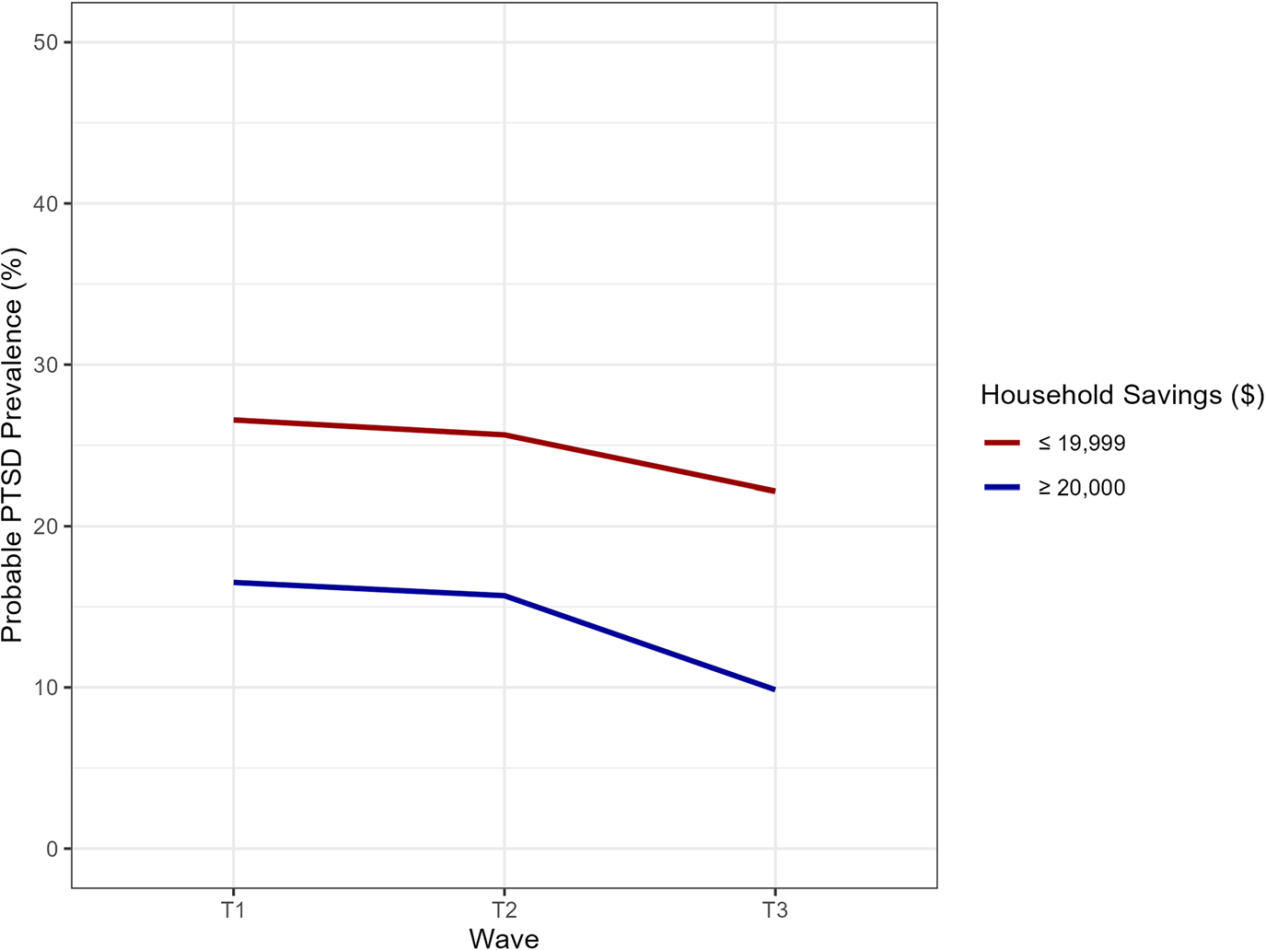
Prevalence of probable PTSD by savings level and data collection wave, anchored savings to T1. Household savings data anchored to T1. Respondents have same household savings in T2 and T3 as they did in T1. Household income in $USD. Data weighted. T1 weights used for T1 estimates; T2 weights used for T2 estimates; T3 weights used for T3 estimates. Probable PTSD defined by Primary Care PTSD Screen for DSM-5 (PC-PTSD-4) score of 3 or greater. Data source: COVID-19 and Life Stressors Impact on Mental Health and Well-being study. Time 1 collected from March 31, 2020, to April 13, 2020. Time 2 collected from March 23, 2021, to April 19, 2021. Time 3 collected from March 22, 2022, to April 18, 2022. At T1, 32 respondents had missing or unknown household savings. At T2, 38 respondents had missing or unknown household savings. At T3, 33 respondents had missing or unknown household savings.

Over a quarter of the group with $19,999 or less in household savings had probable PTSD at T1 and T2, decreasing to under a quarter at T3. In the group with more than $20,000 in household savings, 16.5% had probable PTSD at T1, 16.4% at T2, and 11.0% at T3. The relation between household savings and probable PTSD was statistically significant. Figure 2 shows the probable PTSD prevalence at each time point, stratified by household savings level anchored to T1. Participants with $19,999 or less in household savings at T1 had a higher prevalence of probable PTSD than participants with more in household savings at any time point. Supplementary Fig. 2 includes error bars.

**Fig. 3:**
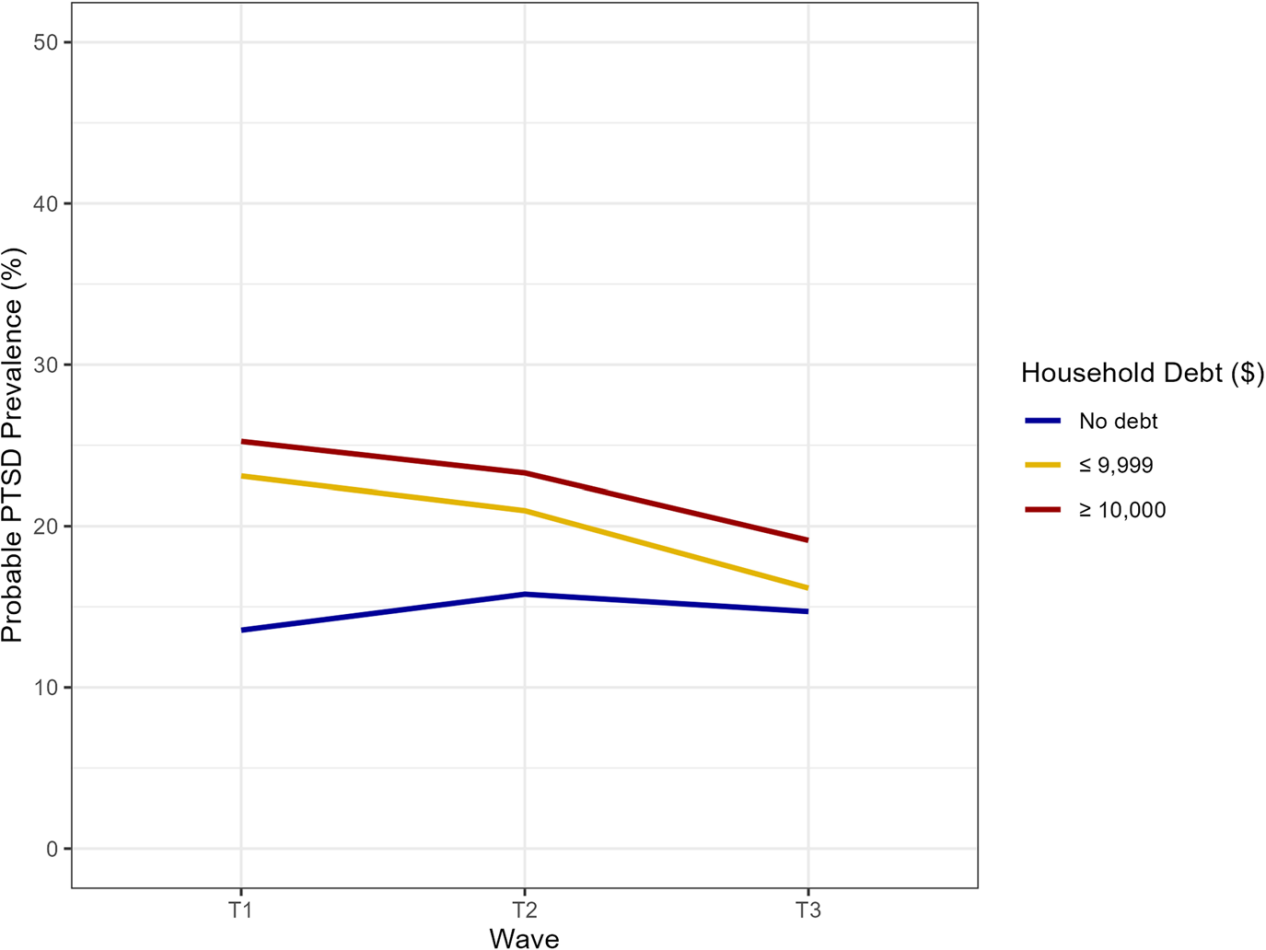
Prevalence of probable PTSD by debt level and data collection wave, anchored debt to T1. Household debt data anchored to T1. Respondents have the same household debt as they did in T1. Household debt in $USD. Data weighted. T1 weights used for T1 estimates; T2 weights used for T2 estimates; T3 weights used for T3 estimates. Probable PTSD defined by Primary Care PTSD Screen for DSM-5 (PC-PTSD-4) score of 3 or greater. Data source: COVID-19 and Life Stressors Impact on Mental Health and Well-Being study. Time 1 collected from March 31, 2020, to April 13, 2020. Time 2 collected from March 23, 2021, to April 19, 2021. Time 3 collected from March 22, 2022, to April 18, 2022. At T1, 21 respondents had missing or unknown household debt. At T2, 27 respondents had missing or unknown household debt. At T3, 24 respondents had missing or unknown household debt.

Over a quarter of the group with $10,000 or more in household debt had probable PTSD at T1, decreasing to under a fifth at T3. The group with $9999 or less in household debt paralleled this trend, decreasing from 23.1% at T1 to 16.2% at T3. Participants with no debt had the lowest probable PTSD at every time point, reaching as low as 13.6% at T1, increasing to 15.8% at T2, and decreasing to 14.7% at T3. Figure 3 shows the probable PTSD prevalence at each time point. Supplementary Fig. 3 includes error bars.

More than a fifth of renters had probable PTSD, with more having probable PTSD at each time point compared to homeowners. Participants in the other (non-home owners or renters) category had the highest rate of probable PTSD. There was a statistically significant relation between home ownership type and probable PTSD at T3. The mean household size of participants with probable PTSD decreased in each subsequent year. At T1, the mean household size was larger in participants with probable PTSD compared to the mean household size in participants without probable PTSD (Table 1).

### Pandemic-related stressors and probable PTSD

**Fig. 4:**
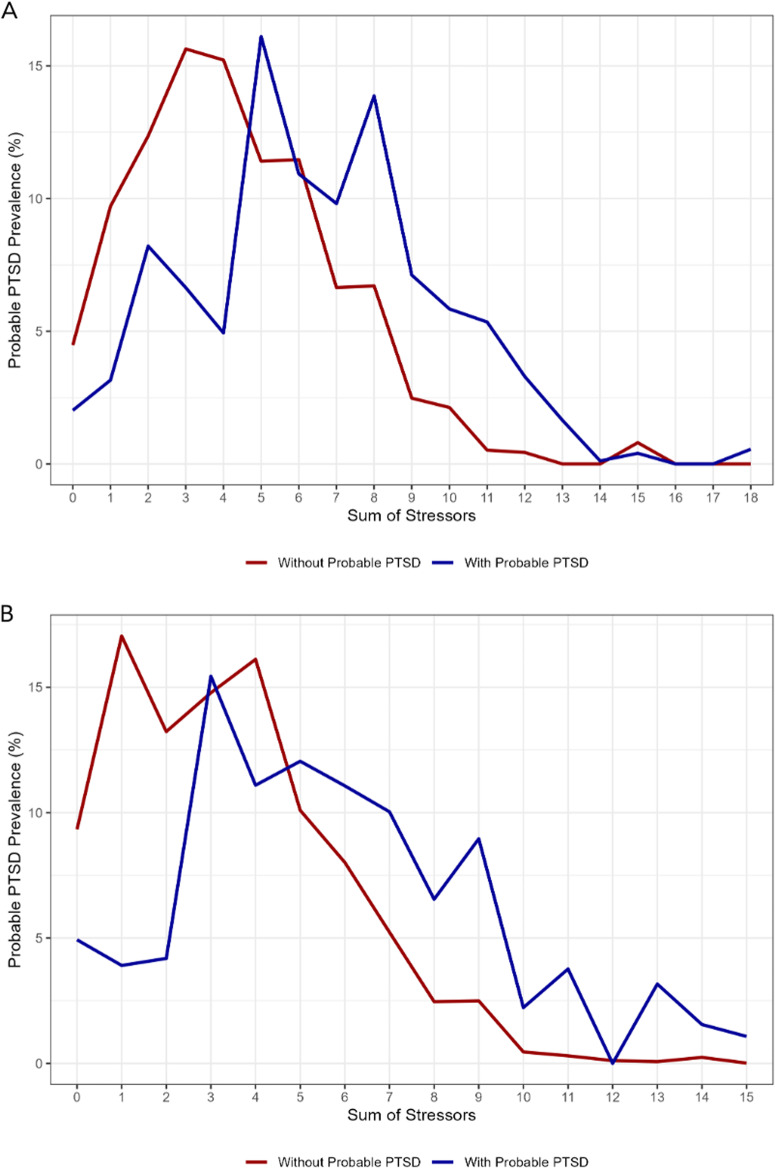
Distribution of Covid-19 Stressors by probable PTSD status at different times during the pandemic. **A** Distribution of Covid-19 Stressors by probable PTSD status at wave T2. **B** Distribution of Covid-19 Stressors by probable PTSD status at wave T3. **A** T2 weights used to calculate probable PTSD prevalence. Stressors defined by presence of: having an event canceled due to the Covid-19 pandemic, seeing friends in person less, seeing family in person less, experiencing travel restrictions, experiencing the death of someone close to you due to Covid-19, having family or relationship problems, having challenges finding childcare, feeling alone, not being able to get food due to shortages, not being able to get supplies due to shortages, losing a job, member of household losing a job, having financial problems, and having difficulty paying rent. Probable PTSD defined by Primary Care PTSD Screen for DSM-5 (PC-PTSD-4) score of 3 or greater. Data source: COVID-19 and Life Stressors Impact on Mental Health and Well-being study. Time 3 collected from March 22, 2022, to April 18, 2022. **B** T3 weights used to calculate probable PTSD prevalence. Stressors defined by presence of: having an event canceled due to the Covid-19 pandemic, seeing friends in person less, seeing family in person less, experiencing travel restrictions, experiencing the death of someone close to you due to Covid-19, having family or relationship problems, having challenges finding childcare, feeling alone, not being able to get food due to shortages, not being able to get supplies due to shortages, losing a job, member of household losing a job, having financial problems, and having difficulty paying rent. Probable PTSD defined by Primary Care PTSD Screen for DSM-5 (PC-PTSD-4) score of 3 or greater. Data source: COVID-19 and Life Stressors Impact on Mental Health and Well-being study. Time 3 collected from March 22, 2022, to April 18, 2022.

At each time point, approximately a third of participants with a high stressor score had probable PTSD. Supplementary Table 4 provides the distribution of stressor scores for each wave. Approximately a fifth of participants with a medium number of stressors had probable PTSD. Around a tenth of participants who had a low number of stressors had probable PTSD. The association between stressor category and probable PTSD was statistically significant. Figure 4A shows the probable PTSD prevalence for each stressor sum at T2. Figure 4B shows the probable PTSD prevalence for each stressor sum at T3. At both timepoints, participants with probable PTSD reported a higher number of stressors than participants without probable PTSD.

**Fig. 5:**
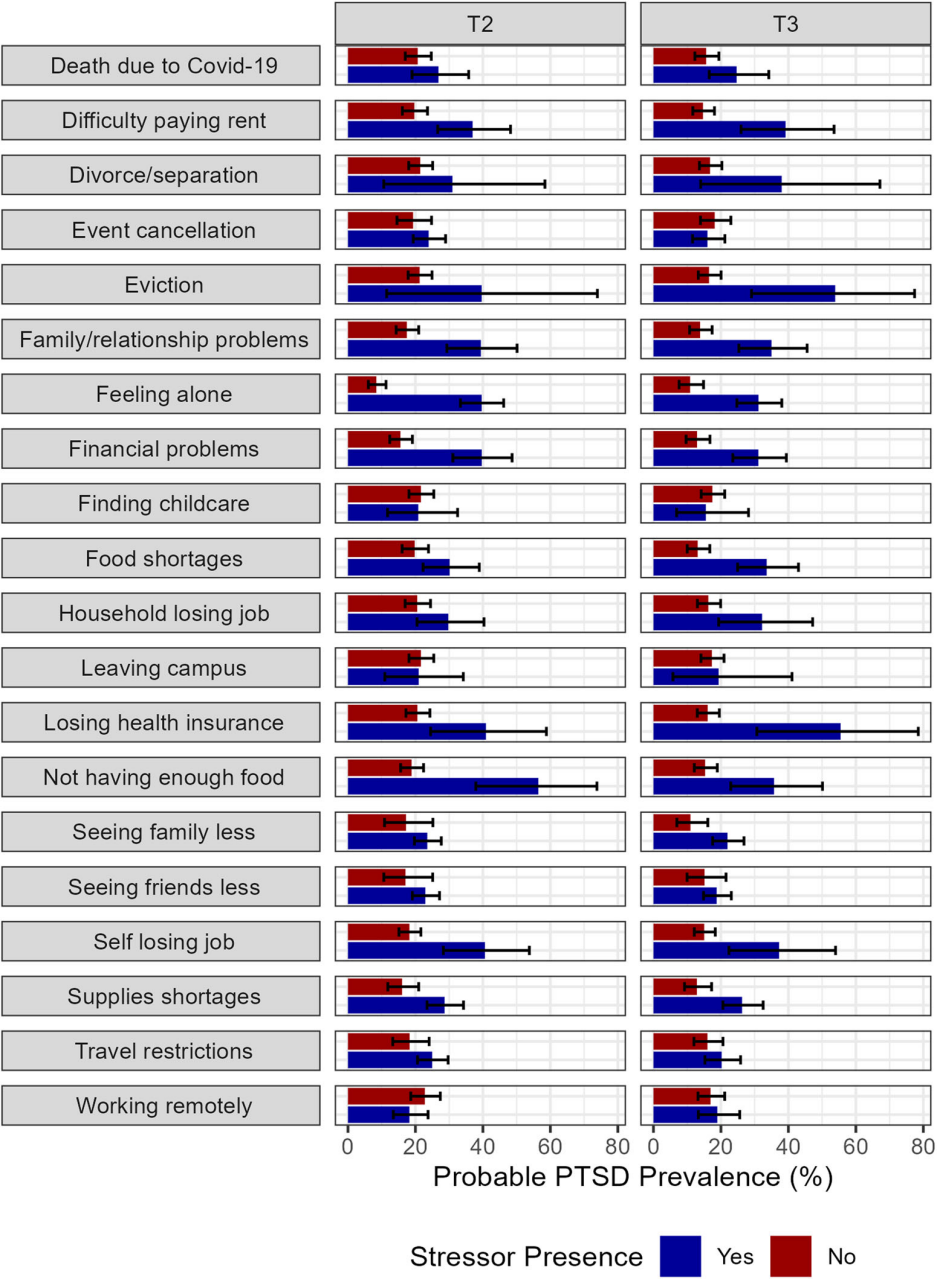
Probable PTSD prevalence by presence of stressor at T2 and T3. T2 weights used to calculate T2 variables. T3 weights used to calculate T3 variables. Stressors defined by presence of: having an event canceled due to the Covid-19 pandemic, seeing friends in person less, seeing family in person less, experiencing travel restrictions, experiencing the death of someone close to you due to Covid-19, having family or relationship problems, having challenges finding childcare, feeling alone, not being able to get food due to shortages, not being able to get supplies due to shortages, losing a job, member of household losing a job, having financial problems, having difficulty paying rent, being forced to leave campus, experiencing eviction or lost housing, not having enough food to eat, losing health insurance, or experiencing divorce or partner separation. Probable PTSD defined by Primary Care PTSD Screen for DSM-5 (PC-PTSD-4). Data source: COVID-19 and Life Stressors Impact on Mental Health and Well-being study. Time 2 collected from March 23, 2021, to April 19, 2021. Time 3 collected from March 22, 2022, to April 18, 2022. Error bars represent 95% confidence intervals calculated using a Rao-Scott correction.

Figure 5 shows the prevalence of probable PTSD for persons experiencing and not experiencing each stressor at T2 and T3. The prevalence at T1 was presented in a prior publication^3^. At T2, there was a greater prevalence of probable PTSD in persons who had experienced most stressors, except for having difficulty finding childcare, being forced to leave campus, and working remotely. Over half of persons who experienced not having enough food had probable PTSD (56.4% (95% CI: 37.9%, 73.8%)). Less than a fifth of persons who experienced working remotely had probable PTSD (18.2% (95% CI: 13.4%, 23.7%)). At T3, there was a greater prevalence of probable PTSD in persons who had experienced most stressors, except for event cancellation and difficulty finding childcare. Of persons who lost health insurance, 55.5% had probable PTSD (95% CI: 30.7%, 78.5%). Having difficulty finding childcare had the lowest prevalence, at 15.5% (95% CI: 6.9%, 28.2%). Supplementary Fig. 4a and 4b show the T2 and T3 prevalence of each PC-PTSD-4 symptom for persons experiencing and not experiencing each stressor.

### Odds ratios of probable PTSD by demographic variables, assets, and stressors

Table 2 shows the adjusted odds of probable PTSD at any given timepoint by the demographic variables, assets, and stressors. Income, home ownership, and stressor score were statistically significant in the model. Persons with $19,999 or less in household income had 2.17 times the odds (95% CI: 1.35, 3.50) and persons with $20,000–$44,999 had 1.63 times the odds (95% CI: 1.14, 2.34) of probable PTSD at any given timepoint relative to persons in households earning $75,000 or more. Persons who rented or had other housing had greater odds of probable PTSD compared to those who owned a home. Persons with a medium stressor count (4–5 Covid-19 stressors) had 1.42 times the odds (95% CI: 1.06, 1.89) and persons with a high stressor count (6+ stressors) had 2.33 times the odds (95% CI: 1.72, 3.15) of probable PTSD relative to persons reporting a low stressor count (0–3 stressors).

**Table 2:** Relation between demographic variables, assets, and probable PTSD at any time during the Covid-19 pandemic

	Odds Ratios [95% CI]	*p* value
Characteristics		
Gender		
Men	1 [Reference]	
Women	1.23 [0.89, 1.71]	0.20
Age		
18–39	1 [Reference]	
40–59	1.0 [0.70, 1.41]	0.98
60+	0.89 [0.54, 1.44]	0.63
Ethnicity		
Asian, non-Hispanic	0.87 [0.34, 2.22]	0.77
Black, non-Hispanic	0.84 [0.50, 1.40]	0.49
Hispanic	1.16 [0.71, 1.88]	0.56
Multiple or other	0.74 [0.43, 1.26]	0.27
White, non-Hispanic	1 [Reference]	
Educational attainment		
Less than a college degree	1.10 [0.80, 1.53]	0.56
College degree or more	1 [Reference]	
Income		
≤19,999	2.17 [1.35, 3.50]	<0.01
20,000–44,999	1.63 [1.14, 2.34]	<0.01
45,000–74,999	1.26 [0.88, 1.82]	0.21
≥75,000	1 [Reference]	
Savings		
≤19,999	1.07 [0.77, 1.49]	0.69
≥20,000	1 [Reference]	
Debt		
No debt	1 [Reference]	
≤9999	1.13 [0.80, 1.61]	0.49
≥10,000	1.65 [0.90, 3.03]	0.11
Home ownership		
Own	1 [Reference]	
Rent	1.64 [1.11, 2.44]	0.01
Other	2.02 [1.33, 3.07]	<0.01
Household size (mean)	1.02 [0.93, 1.13]	0.63
Covid-19 infection		
Did not have Covid-19 infection	1 [Reference]	
Had Covid-19 infection	1.12 [0.73, 1.72]	0.60
Covid-19 vaccine		
Did not have Covid-19 vaccine	1 [Reference]	
Had Covid-19 vaccine	1.17 [0.94, 1.46]	0.16
Stressor Category		
Low (0–3)	1 [Reference]	
Medium (4–5)	1.42 [1.06, 1.89]	0.02
High (6+)	2.33 [1.72, 3.15]	<0.01

Generalized estimating equation (GEE) used to account for repeated measurements of individuals over time. Adjusted model controlled for all variables in table.

Data weighted using weights of participants who had a PTSD score for at least two waves

Probable PTSD defined by Primary Care PTSD Screen for DSM-5 (PC-PTSD-4) score of 3 or greater.

Data source: COVID-19 and Life Stressors Impact on Mental Health and Well-being study. Time 1 collected from March 31, 2020, to April 13, 2020. Time 2 collected from March 23, 2021, to April 19, 2021. Time 3 collected from March 22, 2022, to April 18, 2022.

*N* = 1159 unique groups measured at T1, *N* = 1070 unique groups measured at T2, and *N* = 954 unique groups measured at T3 in the full model.

Table 3 shows the adjusted odds of probable PTSD at T1, T2, and T3 across demographics, assets, and stressors. For each demographic level, the greatest odds of probable PTSD generally occurred at T2. Women had greater odds of probable PTSD at all timepoints relative to men, but the difference in odds decreased in T2 and T3. The 40–59 age category had greater odds than the 18–39 age category at all time points, while the 60+ category had lower odds than the 18–29 age category at all time points. Persons with a history of Covid-19 infection had greater odds of probable PTSD at any time point. Persons with at least one Covid-19 vaccine dose had greater odds of probable PTSD at T2 (1.92 (95% CI: 1.19, 3.11)) and T3 (2.44 (95% CI: 1.29, 4.62)), and these odds were statistically significant.

**Table 3:** Adjusted associations among key assets, stressors, and probable PTSD at T1 (March-April 2020), T2 (March-April 2021), and T3 (March-April 2022)

	2020 (T1)		2021 (T2)		2022 (T3)	
	Odds Ratios [95% CI]	*p* value	Odds Ratios [95% CI]	*p* value	Odds Ratios [95% CI]	*p* value
Characteristics						
Gender						
Men	1 [Reference]		1 [Reference]		1 [Reference]	
Women	1.47 [1.00–2.16]	0.05	1.14 [0.71–1.83]	0.5945	1.04 [0.61–1.80]	0.88
Age						
18–39	1 [Reference]		1 [Reference]		1 [Reference]	
40–59	1.05 [0.68–1.63]	0.84	1.11 [0.66–1.88]	0.69	1.25 [0.70–2.23]	0.44
60+	0.63 [0.33–1.20]	0.16	0.94 [0.44–1.99]	0.87	0.90 [0.41–1.98]	0.80
Ethnicity						
Asian, non-Hispanic	0.47 [0.12–1.85]	0.28	0.59 [0.13–2.72]	0.50	1.68 [0.45–6.22]	0.44
Black, non-Hispanic	0.70 [0.36–1.36]	0.29	1.20 [0.60–2.40]	0.61	1.06 [0.48–2.35]	0.89
Hispanic	1.26 [0.73–2.19]	0.41	1.58 [0.80–3.09]	0.19	1.05 [0.47–2.37]	0.90
Multiple or other	0.44 [0.19–1.03]	0.06	0.66 [0.23–1.92]	0.45	0.80 [0.31–2.04]	0.63
White, non-Hispanic	1 [Reference]		1 [Reference]		1 [Reference]	
Educational attainment						
Less than a college degree	1.04 [0.70–1.56]	0.84	0.82 [0.51–1.32]	0.42	1.36 [0.79–2.37]	0.27
College degree or more	1 [Reference]		1 [Reference]		1 [Reference]	
Income						
≤19,999	2.05 [1.11–3.77]	0.02	2.89 [1.30–6.43]	<0.01	2.39 [1.00–5.71]	0.05
20,000–44,999	0.86 [0.50–1.48]	0.58	3.08 [1.49–6.38]	<0.01	1.31 [0.66–2.61]	0.44
45,000–74,999	0.86 [0.51–1.47]	0.58	1.84 [0.96–3.52]	0.07	1.43 [0.69–2.99]	0.34
≥75,000	1 [Reference]		1 [Reference]		1 [Reference]	
Savings						
≤19,999	1.21 [0.76–1.94]	0.42	1.37 [0.75–2.49]	0.31	1.41 [0.72–2.74]	0.32
≥20,000	1 [Reference]		1 [Reference]		1 [Reference]	
Debt						
No debt	1 [Reference]		1 [Reference]		1 [Reference]	
≤9999	0.87 [0.56–1.33]	0.52	0.87 [0.55–1.39]	0.56	1.29 [0.72–2.32]	0.40
≥10,000	0.98 [0.41–2.37]	0.97	2.06 [0.67–6.27]	0.20	4.18 [1.84–9.52]	<0.01
Home ownership						
Own	1 [Reference]		1 [Reference]		1 [Reference]	
Rent	1.71 [0.92–3.18]	0.09	0.96 [0.52–1.77]	0.89	2.25 [0.90–5.62]	0.08
Other	2.12 [1.17–3.85]	0.01	1.81 [0.98–3.32]	0.06	2.04 [0.81–5.14]	0.13
Household size (mean)	1.00 [0.88–1.13]	0.99	1.00 [0.85–1.18]	0.99	0.91 [0.77–1.06]	0.23
Covid-19 infection						
Did not have Covid-19 infection	1 [Reference]		1 [Reference]		1 [Reference]	
Had Covid-19 infection	8.63 [1.80–41.4]	<0.01	1.56 [0.75–3.24]	0.23	1.16 [0.67–1.99]	0.59
Covid-19 vaccine						
Did not have Covid-19 vaccine	NA		1 [Reference]		1 [Reference]	
Had Covid-19 vaccine	NA	NA	1.92 [1.19–3.11]	<0.01	2.44 [1.29–4.62]	<0.01
Stressor Category						
Low (0–3)	1 [Reference]		1 [Reference]		1 [Reference]	
Medium (4–5)	1.64 [0.88–3.05]	0.12	1.80 [0.94–3.46]	0.08	2.22 [1.13–4.36]	0.02
High (6+)	2.69 [1.56–4.66]	<0.01	4.58 [2.52–8.30]	<0.01	3.89 [2.05–7.38]	<0.01

Models adjusted for gender, age, ethnicity, educational attainment, household income, household savings, home ownership, and mean household size, history of COVID-19 infection, and history of COVID-19 vaccine.

Time 1 weights used for Time 1 analyses; Time 2 weights used for Time 2 analyses; Time 3 weights used for Time 3 analyses.

Probable PTSD defined by Primary Care PTSD Screen for DSM-5 (PC-PTSD-4) score of 3 or greater.

Data source: COVID-19 and Life Stressors Impact on Mental Health and Well-being study. Time 1 collected from March 31, 2020 to April 13, 2020. Time 2 collected from March 23, 2021 to April 19, 2021. Time 3 collected from March 22, 2022 to April 18, 2022.

Time 1, *N* = 1203; Time 2, *N* = 1071; Time 3, *N* = 952.

Covariates collected at Time 1 were used for Time 1 estimates; covariates collected at Time 2 were used for Time 2 estimates. Covariates collected at Time 3 were used for Time 3 estimates.

At T1, 26 participants were missing household income, 32 participants were missing household savings, one participant was missing data for COVID-19 infection, and nine participants were missing stressors. At T2, 38 participants were missing household income, 47 participants were missing household savings, and 43 participants were missing stressors. At T3, 32 participants were missing household income, 46 participants were missing household savings, one participant was missing data for COVID-19 infection, 11 participants were missing data for COVID-19 vaccination, and 79 participants were missing stressors.

Persons in the lowest household income group ($19,999 or less) had the greatest odds of probable PTSD at T1 (2.05 (95% CI: 1.11, 3.77)) and T3 (2.39, 95% CI: 1.00, 5.71). At T2, persons in households earning $20,000 to $44,999 had the highest odds of probable PTSD compared to the highest-earning group (3.08 (95% CI: 1.49, 6.38)), and these odds were statistically significant. Persons with $19,999 or less in household savings had greater odds of probable PTSD than persons with $20,000 or more in household savings, at any time point (Table 3). Persons with $10,000 or more in household debt had the highest odds of PTSD at T3 (4.18 (95% CI: 1.84, 9.52)).

Compared to persons with a low stressor score, persons with a medium stressor score had greater odds of probable PTSD at T1 (1.64 (95% CI: 0.88, 3.05)), T2 (1.80 (95% CI: 0.94, 3.46)), and T3 (2.22 (95% CI: 1.13, 4.36)), with the odds at T3 reaching statistical significance. Persons with a high stressor score had statistically significantly greater odds of probable PTSD at T1 (2.69 (95% CI: 1.56, 4.66)), T2 (4.58 (95% CI: 2.52, 8.30)), and T3 (3.89 (95% CI: 2.05, 7.38)) relative to persons with a low number of stressors.

## Discussion

This longitudinal representative survey of adults in the United States showed that the population burden of PTSD decreased three years after the start of the pandemic, while still remaining higher than pre-pandemic estimates. Reductions in probable PTSD were strongest for groups with more assets. Moreover, experiencing a greater number of pandemic-related stressors increased the odds of having probable PTSD throughout the pandemic.

Compared to 2020 we found that the prevalence of probable PTSD did not change substantially in 2021 but decreased in 2022. However, the prevalence continued to be higher than pre-pandemic estimates^47,48^. Our findings are consistent with analyses from other mass traumatic events^20^, and other studies during the Covid-19 pandemic. Chi et al., showed a decline in PTSD prevalence six months into the pandemic in China, but the prevalence continued to be greater than pre-pandemic levels^49^. Similarly, over a longer term, Benatov et al., reported decreases in PTSD prevalence in four countries between February and June of 2021^50^.

This study found that lack of access to assets, particularly having low income when the pandemic began, was associated with probable PTSD throughout the pandemic. Access to fewer assets—including income, savings, and home ownership—has been associated with a higher burden of mental health indicators, including PTSD, during the pandemic^15,51–53^. For example, in a large cross-sectional sample of US adults, Zhu and colleagues reported similar findings for respondents with low income; those earning $75,000 or less had a higher prevalence of PTSD symptoms (24.0%) than those earning $75,000 to $149,999 (21.2%), and those earning $150,000 and over had the lowest prevalence of PTSD symptoms (17.9%)^54^. Our study adds to the literature by showing the association between low income at the beginning of the pandemic and persistent probable PTSD two years later.

Our analysis showed that experiencing Covid-19 stressors was associated with higher likelihood of probable PTSD throughout the pandemic. This is consistent with previous work that showed that experiencing more pandemic-related stressors was associated with a greater likelihood of depression^13^. Conversely, Zaken and colleagues found that when controlling for demographic factors and PTSD history, the association of Covid-19-related stressors was not associated with PTSD^24^. This divergence could potentially be due to differences in the type of stressors examined as well as tools used to assess PTSD.

We also found that experiencing specific social or economic stressor was generally associated with higher PTSD prevalence. Similarly Zaken et al., reported higher post-traumatic stress among participants who reported loss of health insurance coverage, housing-related problems, financial difficulties, and difficulty accessing food or vital supplies^24^. In a longitudinal study by Ochnik et al., worsening economic status was associated with 1.8 times greater odds of PTSD during the pandemic^55^. Our analysis particularly highlighted the association between experiencing food insecurity and greater likelihood of probable PTSD. This is consistent with two studies from Spain and Bangladesh that reported similar results earlier in the pandemic^56,57^. Conversely, Liddell et al., reported associations between stressors related to Covid-19 infection but not to social stressors, access to support, or trusting authority and PTSD among refugees in Australia^58^. This variance can potentially be due to the different contexts, which can either heighten or minimize the role of economic and social stressors on mental health.

This analysis is not without limitations. First, as with any large panel study, there is the risk of loss to follow-up. Of the 1470 participants who completed T1, 1002 completed all three waves with a complete PTSD score (68.2%). This was largely due to the strict criteria we implemented on which participants to include in the analysis. Increasing the completion status criterion to two or more waves reduces the loss to follow-up, producing a response rate of 85.6%. Second, the lack of data from participants before the pandemic limits direct comparison to PTSD prevalence prior to the pandemic. Third, participants’ historical diagnoses of probable PTSD were not included in the analysis. However, this analysis does provide a representative, longitudinal, understanding of pandemic stressors and probable PTSD during the pandemic.

Notwithstanding these limitations, these findings demonstrate the long-term impact of the pandemic on population mental health. Over the past three years, experiencing and recovering from PTSD differed by access to assets, with those with fewer economic assets bearing the brunt of the burden. Moreover, exposure to more pandemic-related stressors was associated with higher PTSD prevalence throughout the pandemic. Inequities prior to, and during, the pandemic produced health disparities in population mental health outcomes. Reducing these mental health disparities requires addressing asset inequities as well as take mental health consequences as a consideration when designing efforts to mitigate the spread of disease outbreaks.

### Supplementary information

Supplementary

## Data Availability

De-identifiable data are available upon reasonable request made to the corresponding author.

## Code availability

R code is available upon request.

## References

[1] Murata S (2021). The psychiatric sequelae of the COVID‐19 pandemic in adolescents, adults, and health care workers. Depress Anxiety.

[2] Young, K.S. et al. Depression, anxiety and PTSD symptoms before and during the COVID-19 pandemic in the UK. *Psychol. Med*. 1–14. 10.1017/S0033291722002501 (2022). 10.1017/S0033291722002501 PMC10482709 35879886

[3] Abdalla SM, Ettman CK, Cohen GH, Galea S (2021). Mental health consequences of COVID-19: a nationally representative cross-sectional study of pandemic-related stressors and anxiety disorders in the USA. BMJ Open.

[4] Galea S (2002). Psychological sequelae of the september 11 terrorist attacks in New York City. N Engl. J. Med..

[5] Galea S, Tracy M, Norris F, Coffey SF (2008). Financial and social circumstances and the incidence and course of PTSD in Mississippi during the first two years after Hurricane Katrina. J. Trauma Stress.

[6] Ettman CK (2022). Persistent depressive symptoms during COVID-19: a national, population-representative, longitudinal study of U.S. adults. Lancet Reg. Health Am.

[7] Jalloh MF (2015). Impact of Ebola experiences and risk perceptions on mental health in Sierra Leone. BMJ Glob. Health.

[8] Betancourt, T. S. et al. Associations between mental health and Ebola-related health behaviors: A regionally representative cross-sectional survey in post-conflict Sierra Leone. Hay PJ, ed. *PLOS Med*. **13**, e1002073 (2016). 10.1371/journal.pmed.1002073 PMC4978463 27505186

[9] Gissurardóttir ÓS, Hlodversdóttir H, Thordardóttir EB, Pétursdóttir G, Hauksdóttir A (2019). Mental health effects following the eruption in Eyjafjallajökull volcano in Iceland: a population-based study. Scand. J. Public Health.

[10] Wickrama KAS, Kaspar V (2007). Family context of mental health risk in Tsunami-exposed adolescents: findings from a pilot study in Sri Lanka. Soc. Sci. Med..

[11] Cao X (2015). Patterns of DSM-5 posttraumatic stress disorder and depression symptoms in an epidemiological sample of Chinese earthquake survivors: a latent profile analysis. J. Affect. Disord..

[12] Hawryluck L (2004). SARS control and psychological effects of quarantine, Toronto, Canada. Emerg Infect. Dis..

[13] Ettman CK (2020). Prevalence of depression symptoms in US adults before and during the COVID-19 pandemic. JAMA Netw. Open.

[14] Islam MS (2021). Financial and mental health concerns of impoverished urban-dwelling Bangladeshi people during COVID-19. Front. Psychol..

[15] Breslau J, Roth EA, Baird MD, Carman KG, Collins RL (2021). A longitudinal study of predictors of serious psychological distress during COVID-19 pandemic. Psychol. Med..

[16] Galea S (2007). Exposure to hurricane-related stressors and mental illness after Hurricane Katrina. Arch. Gen. Psychiatry.

[17] Sharma A, Kar N (2019). Posttraumatic stress, depression, and coping following the 2015 Nepal earthquake: a study on adolescents. Disaster Med. Public Health Prep..

[18] Jeong H (2016). Mental health status of people isolated due to middle east respiratory syndrome. Epidemiol. Health.

[19] Miguel-Tobal JJ (2006). PTSD and depression after the Madrid March 11 train bombings. J. Trauma. Stress.

[20] Schwartz RM, Gillezeau CN, Liu B, Lieberman-Cribbin W, Taioli E (2017). Longitudinal impact of Hurricane Sandy exposure on mental health symptoms. Int. J. Environ. Res. Public Health.

[21] Stuber J, Resnick H, Galea S (2006). Gender disparities in posttraumatic stress disorder after mass trauma. Gend. Med..

[22] Tracy M, Norris FH, Galea S (2011). Differences in the determinants of posttraumatic stress disorder and depression after a mass traumatic event. Depress Anxiety.

[23] Motreff Y (2020). Factors associated with PTSD and partial PTSD among first responders following the Paris terror attacks in November 2015. J. Psychiatr. Res..

[24] Zaken MD, Boyraz G, Dickerson SS (2022). COVID‐19 pandemic‐related stressors and posttraumatic stress: the main, moderating, indirect, and mediating effects of social support. Stress Health.

[25] Ouyang H (2022). The increase of PTSD in front-line health care workers during the COVID-19 pandemic and the mediating role of risk perception: a one-year follow-up study. Transl. Psychiatry.

[26] Lueger-Schuster B, Zrnić Novaković I, Lotzin A (2022). Two years of COVID-19 in Austria—Exploratory longitudinal study of mental health outcomes and coping behaviors in the general population. Int. J. Environ. Res. Public Health.

[27] Rass V (2022). Neurological outcomes 1 year after COVID‐19 diagnosis: a prospective longitudinal cohort study. Eur. J. Neurol..

[28] Van der Hallen R, Godor BP (2022). COVID-19 pandemic-related posttraumatic growth in a small cohort of university students: a 1-year longitudinal study. Psychiatry Res..

[29] Bliese PD (2008). Validating the primary care posttraumatic stress disorder screen and the posttraumatic stress disorder checklist with soldiers returning from combat. J. Consult Clin. Psychol..

[30] R Core Team. R: A language and environment for statistical computing. *R Foundation for Statistical Computing* https://www.R-project.org/ (2021).

[31] Dowle, M, Srinivasan, A. Data.table: Extension of ‘data.frame. https://CRAN.R-project.org/package=data.table (2021).

[32] Müller, K. Here: A simpler way to find your files. https://CRAN.R-project.org/package=here (2020).

[33] Lumley, T. Survey: analysis of complex survey samples. https://cran.rproject.org/web/packages/survey/survey.pdf (2020).

[34] Wickham, H., Miller, E. & Smith, D. Haven: Import and export “SPSS”, “Stata” and “SAS” files. https://CRAN.R-project.org/package=haven (2022).

[35] Iannone, R., Cheng, J. & Schloerke, B. Gt: Easily create presentation-ready display tables. https://CRAN.R-project.org/package=gt (2022).

[36] Sjoberg DD, Whiting K, Curry M, Lavery JA, Larmarange J (2021). Reproducible summary tables with the gtsummary Package. R J..

[37] Wickham H (2019). Welcome to the tidyverse. J. Open Sour. Softw..

[38] Halekoh U, Højsgaard S, Yan J (2006). The R Package geepack for generalized estimating equations. J. Stat. Softw.

[39] Petersen, A. H., McDaniel, L., Ekstrøm, C. & Højsgaard, S. Tools for fitting generalized linear models with clustered observations using generalized estimating equation*s*. https://CRAN.R-project.org/package=geeasy (2022).

[40] McDaniel LS, Henderson NC, Rathouz PJ (2013). Fast pure R implementation of GEE: application of the Matrix package. R J..

[41] Ellis, G. F. & Schneider, B. Srvyr: ’Dplyr’-Like Syntax for summary statistics of survey data. https://CRAN.R-project.org/package=srvyr (2022).

[42] Pessoa, D., Damico, A. & Jacob, G. Convey: estimation of indicators on social exclusion and poverty and its linearization, variance estimation. https://github.com/ajdamico/convey/ (2022).

[43] Hvitfeldt, E. Paletteer: Comprehensive collection of color palettes. https://github.com/EmilHvitfeldt/paletteer (2021).

[44] Arnold, J. B. Ggthemes: Extra themes, scales and geoms for “Ggplot2*”*. https://CRAN.R-project.org/package=ggthemes (2021).

[45] Ekstrøm, C. T. MESS: miscellaneous Esoteric Statistical Scripts. https://CRAN.R-project.org/package=MESS (2022)

[46] Rinker, T. W. & Kurkiewicz, D. Pacman: Package Management for R. http://github.com/trinker/pacman (2018).

[47] Kessler RC, Chiu WT, Demler O, Walters EE (2005). Prevalence, severity, and comorbidity of 12-Month DSM-IV disorders in the National Comorbidity Survey Replication. Arch. Gen. Psychiatry.

[48] Bromet EJ (2017). Post-traumatic stress disorder associated with natural and human-made disasters in the World Mental Health Surveys. Psychol. Med..

[49] Chi X (2021). Posttraumatic stress symptoms among Chinese college students during the COVID-19 pandemic: a longitudinal study. Front. Public Health.

[50] Benatov J, Ochnik D, Rogowska AM, Arzenšek A, Mars Bitenc U (2022). Prevalence and sociodemographic predictors of mental health in a representative sample of young adults from Germany, Israel, Poland, and Slovenia: A longitudinal study during the COVID-19 pandemic. Int. J. Environ. Res. Public Health.

[51] Ettman CK, Cohen GH, Vivier PM, Galea S (2021). Savings, home ownership, and depression in low-income US adults. Soc. Psychiatry Psychiatr. Epidemiol..

[52] Goularte JF (2021). COVID-19 and mental health in Brazil: psychiatric symptoms in the general population. J. Psychiatr. Res..

[53] Alleaume C (2022). Incidence of PTSD in the French population a month after the COVID-19 pandemic-related lockdown: evidence from a national longitudinal survey. BMC Public Health.

[54] Zhu K (2021). COVID-19 related symptoms of anxiety, depression, and PTSD among US adults. Psychiatry Res..

[55] Ochnik D (2021). Exposure to COVID-19 during the first and the second wave of the pandemic and coronavirus-related PTSD Risk among University Students from Six Countries—a repeated cross-sectional study. J. Clin. Med..

[56] Bountress KE (2022). The COVID-19 pandemic impacts psychiatric outcomes and alcohol use among college students. Eur. J. Psychotraumatol..

[57] Khan AH (2020). The impact of COVID-19 pandemic on mental health & wellbeing among home-quarantined Bangladeshi students: a cross-sectional pilot study. J. Affect. Disord..

[58] Liddell BJ (2021). The association between COVID-19 related stressors and mental health in refugees living in Australia. Eur. J. Psychotraumatol..

