# Supplementary material for: Post-traumatic stress disorder during the Covid-19 pandemic: a national, population-representative, longitudinal study of U.S. adults
Source: Npj Ment Health Res. 2024 Apr 10;3:20. doi: 10.1038/s44184-024-00059-w (PMC11006849; doi:10.1038/s44184-024-00059-w)
Supplement: Supplementary file 1 — Supplementary [file 44184_2024_59_MOESM1_ESM.docx]

**Supplementary Table 1: Characteristics of T2 responders and T2 non-responders**

|  | **T2 responders** | **T2 non-responders** |  |
| --- | --- | --- | --- |
|  | **N (%)** | **N (%)** | **p-value** |
| **Characteristics** |  |  |  |
| Total | 1,165 (91.6) | 93 (8.4) |  |
| **Gender** |  |  | 0.45 |
| Men | 591 (92.5) | 48 (7.5) |  |
| Women | 574 (90.7) | 45 (9.3) |  |
| **Age** |  |  | 0.06 |
| 18-39 | 464 (87.6) | 51 (12.4) |  |
| 40-59 | 389 (93.2) | 27 (6.8) |  |
| 60+ | 312 (94.4) | 15 (5.6) |  |
| **Ethnicity** |  |  | 0.04 |
| Asian, non-Hispanic | 29 (98.1) | 2 (1.9) |  |
| Black, non-Hispanic | 96 (82.8) | 17 (17.2) |  |
| Hispanic | 192 (90.5) | 21 (9.5) |  |
| Multiple or other | 59 (92.7) | 4 (7.3) |  |
| White, non-Hispanic | 789 (93.0) | 49 (7.0) |  |
| **Educational attainment** |  |  | 0.07 |
| Less than a college degree | 771 (90.1) | 69 (9.9) |  |
| College degree or more | 394 (94.2) | 24 (5.8) |  |
| **Income** |  |  | 0.44 |
| ≤ 19,999 | 175 (88.3) | 23 (11.7) |  |
| 20,000-44,999 | 283 (90.2) | 26 (9.8) |  |
| 45,000-74,999 | 301 (94.0) | 18 (6.0) |  |
| ≥ 75,000 | 381 (92.2) | 25 (7.8) |  |
| **Savings** |  |  | 0.01 |
| ≤ 19,999 | 640 (89.0) | 67 (11.0) |  |
| ≥ 20,000 | 493 (94.7) | 26 (5.3) |  |
| **Debt** |  |  | 0.76 |
| No debt | 210 (90.9) | 17 (19.1) |  |
| ≤ 9,999 | 433 (90.5) | 34 (9.5) |  |
| ≥ 10,000 | 501 (92.5) | 42 (7.5) |  |
| **Home ownership** |  |  | 0.18 |
| Own | 719 (92.9) | 46 (7.1) |  |
| Rent | 399 (88.1) | 44 (11.9) |  |
| Other | 47 (93.7) | 3 (6.3) |  |
| **Household size (mean)** | 3.2 | 3.1 | 0.72 |
| **Covid-19 infection** |  |  | 0.01 |
| Did not have Covid-19 infection | 1,157 (91.8) | 91 (8.2) |  |
| Had Covid-19 infection | 6 (60.6) | 2 (39.4) |  |
| **Covid-19 vaccine (T3)** |  |  | 0.55 |
| Did not have Covid-19 vaccine | 185 (88.8) | 23 (11.2) |  |
| Had Covid-19 vaccine | 803 (90.7) | 69 (9.3) |  |
| **Stressor Category** |  |  | 0.06 |
| Low (0-3) | 285 (88.3) | 26 (11.7) |  |
| Medium (4-5) | 416 (95.3) | 26 (4.7) |  |
| High (6+) | 456 (90.6) | 38 (9.4) |  |

Note:

(a) Frequencies unweighted; percentages weighted. T1 weights used for all estimates. Chi-square tests of independence used to calculate p-values.

(b) Data source: COVID-19 and Life Stressors Impact on Mental Health and Well-being study. Time 1 collected from March 31, 2020, to April 13, 2020. Time 2 collected from March 23, 2021 to April 19, 2021. Time 3 collected from March 22, 2022, to April 18, 2022.

(c) Covariates collected at Time 1 were used for Time 1 estimates, except for the Covid-19 vaccine covariate. For T2 responders, the Covid-19 vaccine covariate was collected at T2. For T2 non-responders, the Covid-19 vaccine covariate was collected at T3.

(d) At T1, 26 participants were missing household income, 33 participants were missing household savings, 21 participants were missing household debt, one participant was missing COVID-19 infection, and nine participants were missing stressors. At T3, 188 participants were missing data for Covid-19 vaccination.

(e) At T1, six participants were missing data for probable PTSD.

**Supplementary Table 2: Characteristics of T3 responders and T3 non-responders**

|  | **T3 responders** | **T3 non-responders** |  |
| --- | --- | --- | --- |
|  | **N (%)** | **N (%)** | **p-value** |
| **Characteristics** |  |  |  |
| Total | 1,081 (86.8) | 177 (13.2) |  |
| **Gender** |  |  | 0.34 |
| Men | 547 (85.7) | 92 (14.3) |  |
| Women | 534 (87.9) | 85 (12.1) |  |
| **Age** |  |  | 0.02 |
| 18-39 | 422 (82.4) | 93 (17.6) |  |
| 40-59 | 365 (89.7) | 51 (10.3) |  |
| 60+ | 294 (88.9) | 33 (11.1) |  |
| **Ethnicity** |  |  | 0.02 |
| Asian, non-Hispanic | 27 (76.8) | 4 (23.2) |  |
| Black, non-Hispanic | 100 (91.7) | 13 (8.3) |  |
| Hispanic | 163 (79.1) | 50 (20.9) |  |
| Multiple or other | 51 (82.7) | 12 (17.3) |  |
| White, non-Hispanic | 740 (88.8) | 98 (11.2) |  |
| **Educational attainment** |  |  | 0.32 |
| Less than a college degree | 715 (86.0) | 125 (14.0) |  |
| College degree or more | 366 (88.4) | 52 (11.6) |  |
| **Income** |  |  | 0.82 |
| ≤ 19,999 | 163 (84.7) | 35 (15.3) |  |
| 20,000-44,999 | 272 (87.9) | 37 (12.1) |  |
| 45,000-74,999 | 279 (87.6) | 40 (12.4) |  |
| ≥ 75,000 | 346 (87.1) | 60 (12.9) |  |
| **Savings** |  |  | 0.56 |
| ≤ 19,999 | 602 (86.3) | 105 (13.7) |  |
| ≥ 20,000 | 452 (87.7) | 67 (12.3) |  |
| **Home ownership** |  |  | 0.12 |
| Own | 672 (88.4) | 93 (11.6) |  |
| Rent | 369 (83.5) | 74 (16.5) |  |
| Other | 40 (84.3) | 10 (15.7) |  |
| **Debt** |  |  | 0.01 |
| No debt | 205 (91.1) | 22 (8.9) |  |
| ≤ 9,999 | 413 (89.0) | 54 (11.0) |  |
| ≥ 10,000 | 445 (82.8) | 98 (17.2) |  |
| **Household size (mean)** | 3.1 | 3.7 | <0.01 |
| **Covid-19 infection** |  |  | 0.92 |
| Did not have Covid-19 infection | 1,072 (86.8) | 176 (13.2) |  |
| Had Covid-19 infection | 7 (85.4) | 1 (14.6) |  |
| **Covid-19 vaccine (T2)** |  |  | 0.94 |
| Did not have Covid-19 vaccine | 562 (85.8) | 107 (14.2) |  |
| Had Covid-19 vaccine | 432 (85.6) | 70 (14.4) |  |
| **Stressor Category** |  |  | 0.15 |
| Low (0-3) | 277 (90.7) | 34 (9.3) |  |
| Medium (4-5) | 382 (86.5) | 60 (13.5) |  |
| High (6+) | 412 (84.8) | 82 (15.2) |  |

Note:

(a) Frequencies unweighted; percentages weighted. T1 weights used for all estimates. Chi-square tests of independence used to calculate p-values.

(b) Data source: COVID-19 and Life Stressors Impact on Mental Health and Well-being study. Time 1 collected from March 31, 2020, to April 13, 2020. Time 2 collected from March 23, 2021, to April 19, 2021. Time 3 collected from March 22, 2022, to April 18, 2022.

(c) Covariates collected at Time 1 were used for Time 1 estimates, except for the Covid-19 vaccine covariate. For T3 responders, the Covid-19 vaccine covariate was collected at T3. For T3 non-responders, the Covid-19 vaccine covariate was collected at T2.

(d) At T1, 26 participants were missing household income, 33 participants were missing household savings, 21 participants were missing household debt, one participant was missing COVID-19 infection, and nine participants were missing stressors. At T2, 93 participants were missing data for Covid-19 vaccination.

(e) At T1, six participants were missing data for probable PTSD.

**Supplementary Table 3: PTSD score by wave**

|  | **2020 (T1)** | **2021 (T2)** | **2022 (T3)** |
| --- | --- | --- | --- |
|  | **N (%)** | **N (%)** | **N (%)** |
| **Characteristics** |  |  |  |
| Total | 1,252 | 1,165 | 1,081 |
| **PTSD score** |  |  |  |
| 0 | 553 (43.4) | 676 (55.0) | 686 (61.2) |
| 1 | 236 (18.1) | 146 (13.3) | 145 (13.9) |
| 2 | 202 (16.3) | 132 (10.5) | 96 (8.1) |
| 3 | 143 (12.3) | 106 (9.4) | 83 (7.2) |
| 4 | 118 (9.9) | 105 (11.7) | 71 (9.6) |

Note:
(a) Frequencies unweighted; percentages weighted. T1 weights used for T1 estimates. T2 weights used for T2 estimates. T3 weights used for T3 estimates.

(b) Data source: COVID-19 and Life Stressors Impact on Mental Health and Well-being study. Time 1 collected from March 31, 2020, to April 13, 2020. Time 2 collected from March 23, 2021, to April 19, 2021. Time 3 collected from March 22, 2022, to April 18, 2022.

(c) Covariates collected at Time 1 were used for Time 1 estimates. Covariates collected at Time 2 were used for Time 2 estimates. Covariates collected at Time 3 were used for Time 3 estimates.

**Supplementary Table 4: Sum of stressors by wave**

|  | **2020 (T1)** | **2021 (T2)** | **2022 (T3)** |
| --- | --- | --- | --- |
|  | **N (%)** | **N (%)** | **N (%)** |
| **Characteristics** |  |  |  |
| Total | 1,252 | 1,165 | 1,081 |
| **Stressor score** |  |  |  |
| 0 | 7 (0.8) | 8 (0.5) | 15 (1.9) |
| 1 | 53 (3.4) | 93 (8.7) | 152 (16.1) |
| 2 | 107 (8.3) | 129 (12.8) | 138 (13.1) |
| 3 | 143 (11.8) | 150 (13.9) | 170 (17.2) |
| 4 | 218 (18.3) | 172 (14.1) | 154 (15.5) |
| 5 | 223 (17.9) | 162 (13.6) | 140 (12.7) |
| 6 | 178 (14.8) | 131 (11.7) | 87 (9.6) |
| 7 | 155 (11.4) | 100 (9.8) | 58 (5.2) |
| 8 | 73 (5.5) | 80 (6.3) | 39 (4.5) |
| 9 | 38 (2.8) | 44 (3.8) | 27 (2.2) |
| 10 | 27 (2.9) | 32 (2.9) | 7 (0.6) |
| 11 | 13 (2.0) | 6 (0.4) | 6 (0.8) |
| 12 | 4 (0.3) | 13 (1.6) | 4 (0.3) |
| 13 | 3 (0.1) | 1 (0.0) | 4 (0.3) |
| 14 | 1 (0.0) | 1 (0.0) | 1 (0.1) |

Note:
(a) Frequencies unweighted; percentages weighted. T1 weights used for T1 estimates. T2 weights used for T2 estimates. T3 weights used for T3 estimates.

(b) Data source: COVID-19 and Life Stressors Impact on Mental Health and Well-being study. Time 1 collected from March 31, 2020, to April 13, 2020. Time 2 collected from March 23, 2021, to April 19, 2021. Time 3 collected from March 22, 2022, to April 18, 2022.

(c) Covariates collected at Time 1 were used for Time 1 estimates. Covariates collected at Time 2 were used for Time 2 estimates. Covariates collected at Time 3 were used for Time 3 estimates.

(d) At T1, nine participants were missing data for stressor score. At T2, 43 participants are missing data for stressor score. At T3, 79 participants were missing data for stressor score.

**Supplementary Figure 1: Prevalence of probable PTSD by income level and data collection wave including error bars, anchored income to T1**


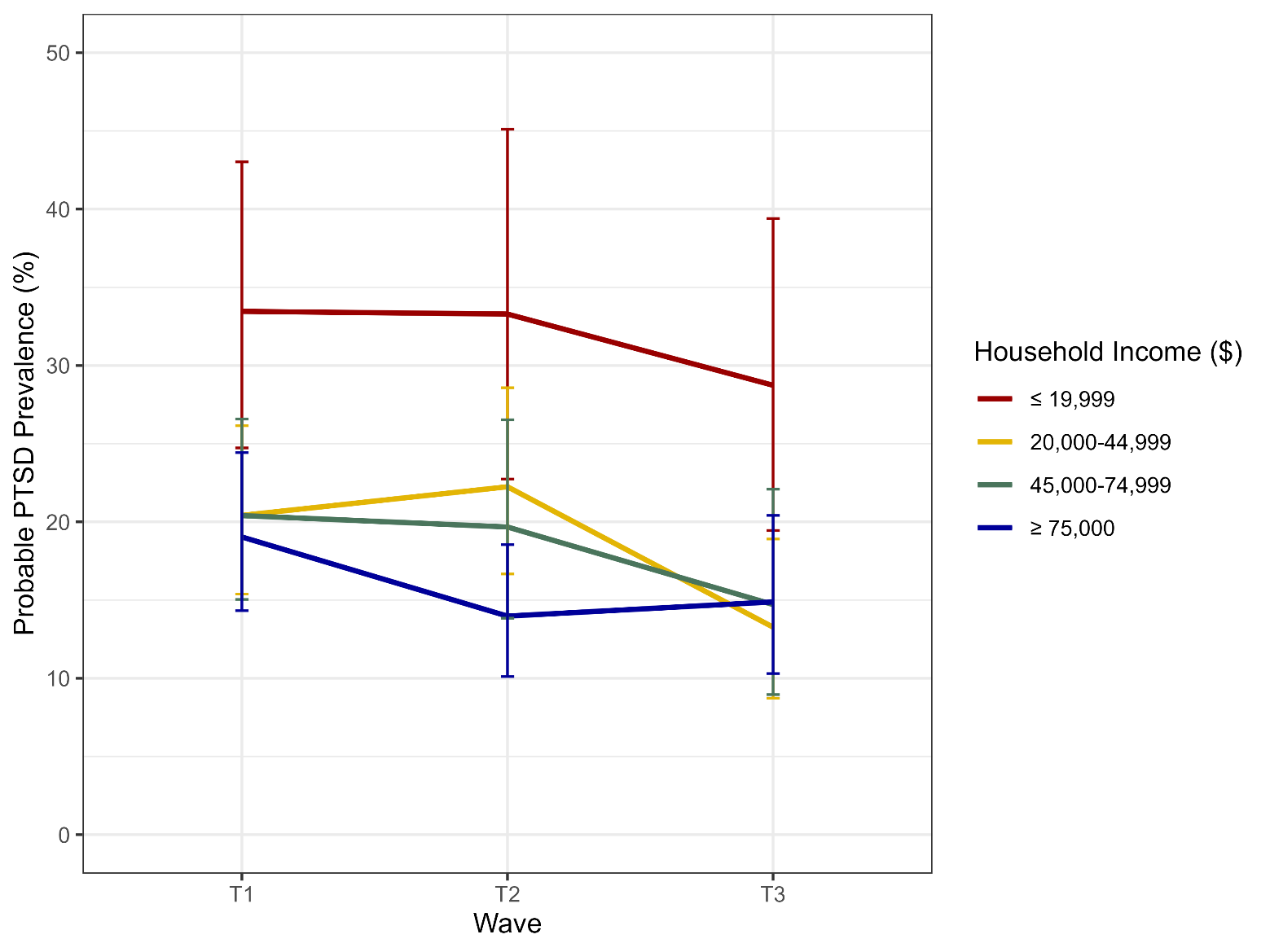


Note:

(a) Household income data anchored to T1. Respondents have the same household income as they did in T1. Household income in $USD.

(b) Data weighted. T1 weights used for T1 estimates; T2 weights used for T2 estimates; T3 weights used for T3 estimates.

(c) Probable PTSD defined by Primary Care PTSD Screen for DSM-5 (PC-PTSD-4) score of 3 or greater.

(d) Data source: COVID-19 and Life Stressors Impact on Mental Health and Well-Being study. Time 1 collected from March 31, 2020, to April 13, 2020. Time 2 collected from March 23, 2021, to April 19, 2021. Time 3 collected from March 22, 2022, to April 18, 2022.

(e) At T1, 26 respondents had missing or unknown household income. At T2, 31 respondents had missing or unknown household income. At T3, 27 respondents had missing or unknown household income.

(f) Error bars represent 95% confidence intervals calculated using a Rao-Scott correction.

**Supplementary Figure 2: Prevalence of probable PTSD by savings level and data collection wave including error bars, anchored savings to T1**


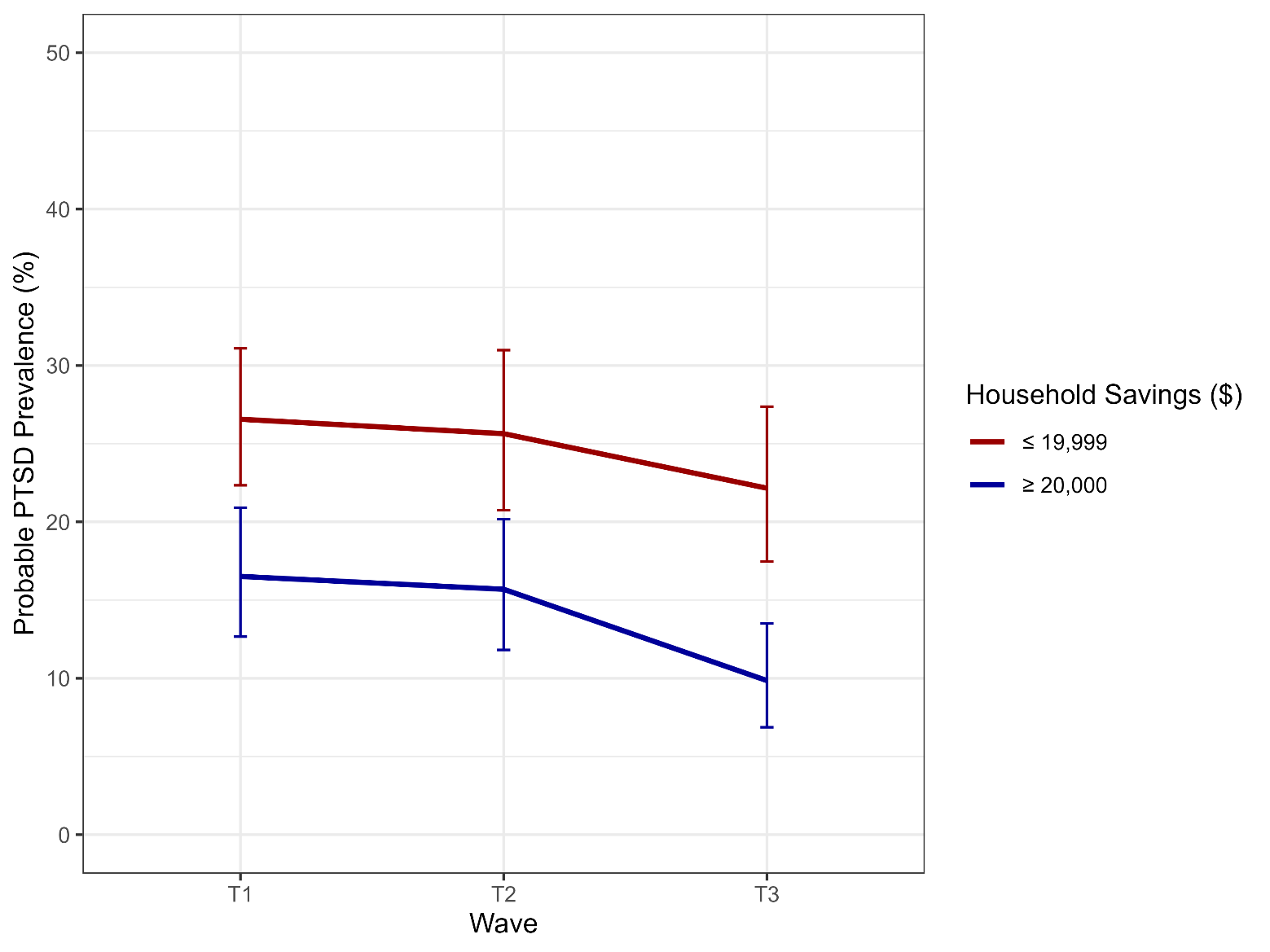


Note:

(a) Household savings data anchored to T1. Respondents have the same household savings as they did in T1. Household savings in $USD.

(b) Data weighted. T1 weights used for T1 estimates; T2 weights used for T2 estimates; T3 weights used for T3 estimates.

(c) Probable PTSD defined by Primary Care PTSD Screen for DSM-5 (PC-PTSD-4) score of 3 or greater.

(d) Data source: COVID-19 and Life Stressors Impact on Mental Health and Well-Being study. Time 1 collected from March 31, 2020, to April 13, 2020. Time 2 collected from March 23, 2021, to April 19, 2021. Time 3 collected from March 22, 2022, to April 18, 2022.

(e) At T1, 32 respondents had missing or unknown household savings. At T2, 38 respondents had missing or unknown household savings. At T3, 33 respondents had missing or unknown household savings.

(f) Error bars represent 95% confidence intervals calculated using a Rao-Scott correction.

**Supplementary Figure 3: Prevalence of probable PTSD by debt level and data collection wave including error bars , anchored debt to T1**


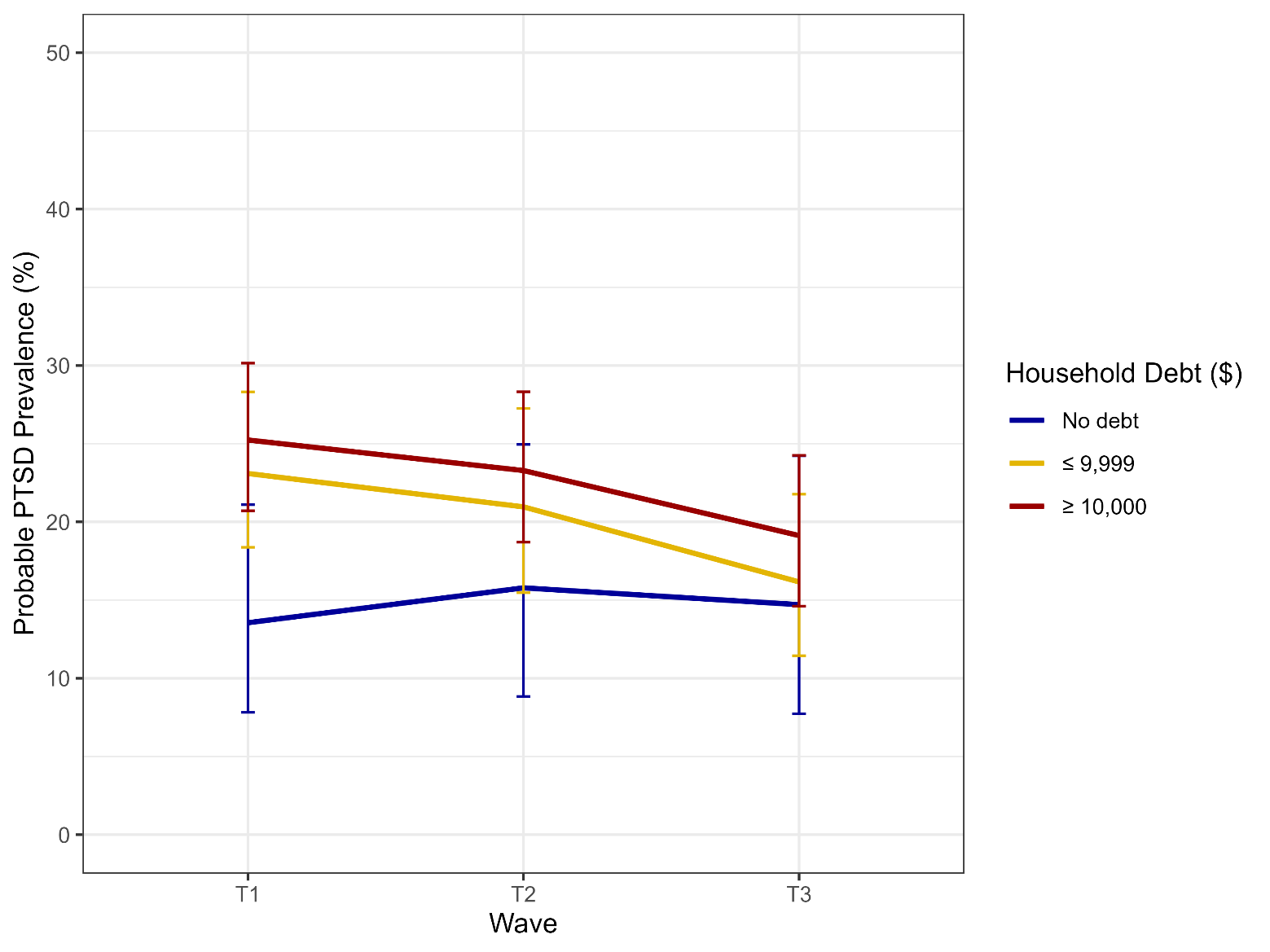


Note:

(a) Household debt data anchored to T1. Respondents have the same household debt as they did in T1. Household debt in $USD.

(b) Data weighted. T1 weights used for T1 estimates; T2 weights used for T2 estimates; T3 weights used for T3 estimates.

(c) Probable PTSD defined by Primary Care PTSD Screen for DSM-5 (PC-PTSD-4) score of 3 or greater.

(d) Data source: COVID-19 and Life Stressors Impact on Mental Health and Well-Being study. Time 1 collected from March 31, 2020, to April 13, 2020. Time 2 collected from March 23, 2021, to April 19, 2021. Time 3 collected from March 22, 2022, to April 18, 2022.

(e) At T1, 21 respondents had missing or unknown household debt. At T2, 27 respondents had missing or unknown household debt. At T3, 24 respondents had missing or unknown household debt.

(f) Error bars represent 95% confidence intervals calculated using a Rao-Scott correction.

**Supplementary Figure 4a: Stressor occurrence by PC-PTSD-4 symptom at T2**


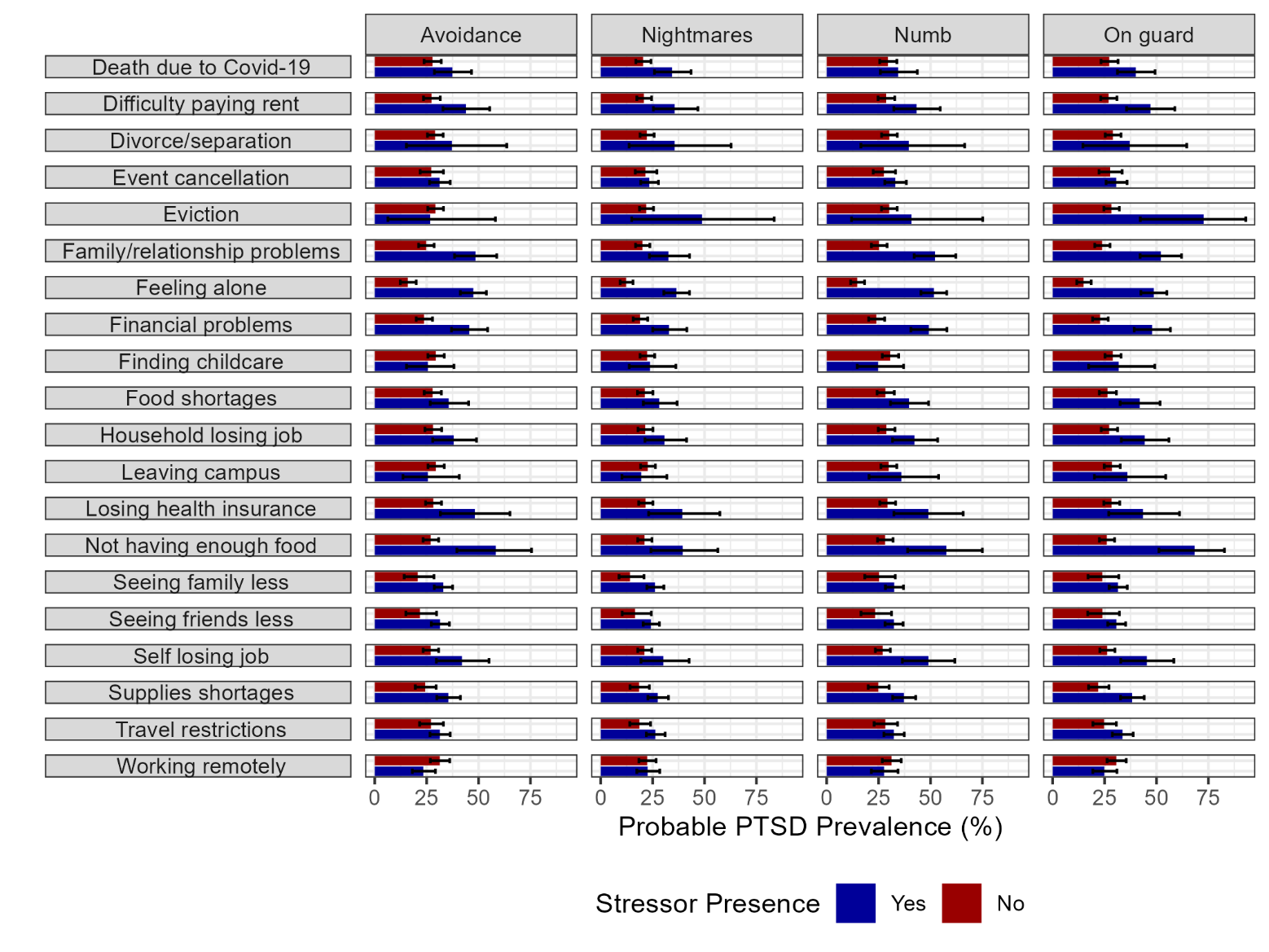


Note:

(a) T2 weights used to calculate weighted percentage of symptoms.

(b) Stressors defined by presence of: having an event cancelled due to the Covid-19 pandemic, seeing friends in person less, seeing family in person less, experiencing travel restrictions, experiencing the death of someone close to you due to Covid-19, having family or relationship problems, having challenges finding childcare, feeling alone, not being able to get food due to shortages, not being able to get supplies due to shortages, losing a job, member of household losing a job, having financial problems, having difficulty paying rent, being forced to leave campus, experiencing eviction or lost housing, not having enough food to eat, losing health insurance, or experiencing divorce or partner separation.

(c) Probable PTSD defined by Primary Care PTSD Screen for DSM-5 (PCL-4).

(d) Data source: COVID-19 and Life Stressors Impact on Mental Health and Well-being study. Time 2 collected from March 23, 2021, to April 19, 2021.

(e) Error bars represent 95% confidence intervals calculated using a Rao-Scott correction.

**Supplementary Figure 4b: Stressor occurrence by PC-PTSD-4 symptom at T3**


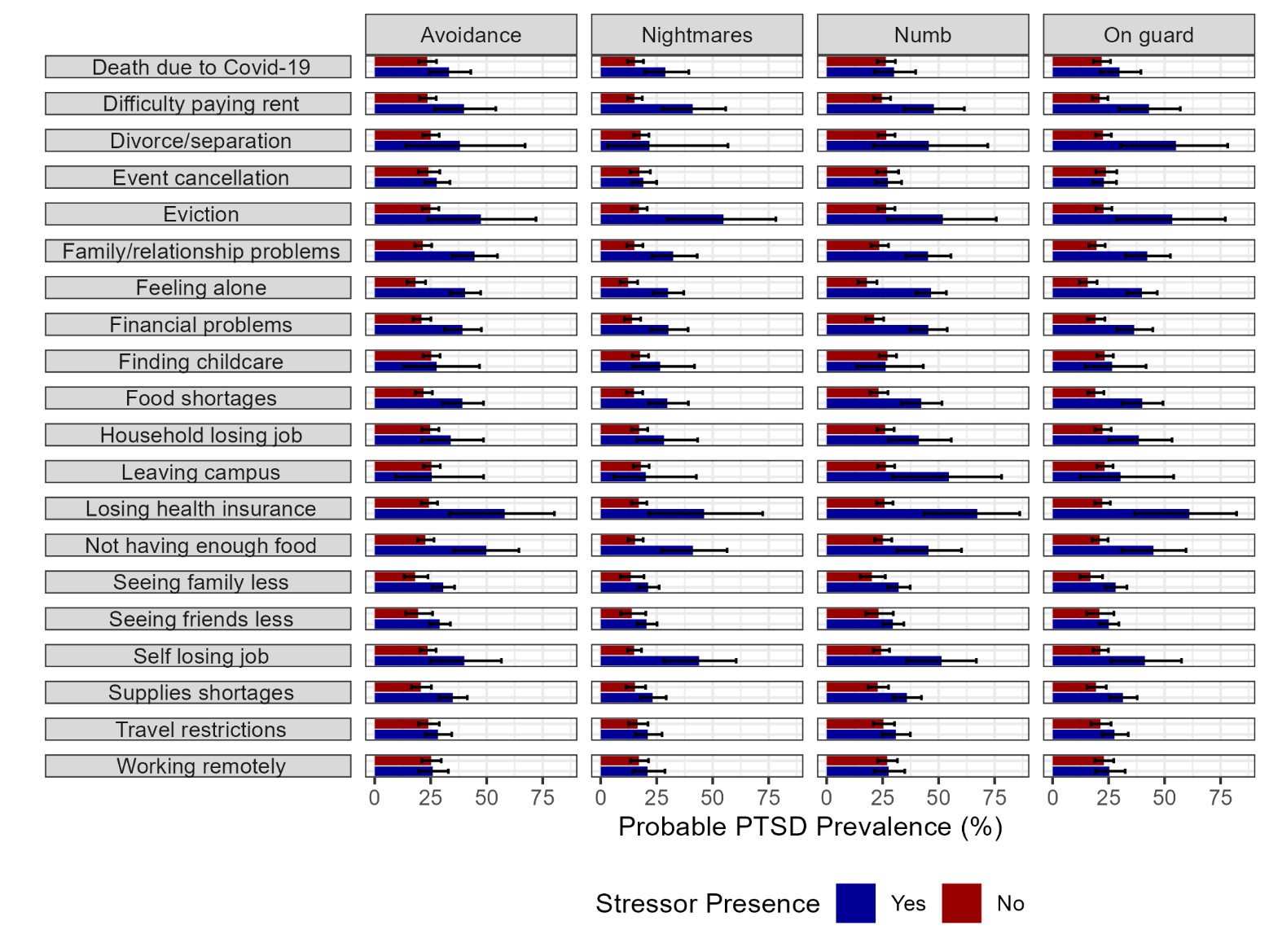


Note:

(a) T3 weights used to calculate weighted percentage of symptoms.

(b) Stressors defined by presence of: having an event cancelled due to the Covid-19 pandemic, seeing friends in person less, seeing family in person less, experiencing travel restrictions, experiencing the death of someone close to you due to Covid-19, having family or relationship problems, having challenges finding childcare, feeling alone, not being able to get food due to shortages, not being able to get supplies due to shortages, losing a job, member of household losing a job, having financial problems, having difficulty paying rent, being forced to leave campus, experiencing eviction or lost housing, not having enough food to eat, losing health insurance, or experiencing divorce or partner separation.

(c) Probable PTSD defined by Primary Care PTSD Screen for DSM-5 (PCL-4).

(d) Data source: COVID-19 and Life Stressors Impact on Mental Health and Well-being study. Time 3 collected from March 22, 2022, to April 18, 2022.

(e) Error bars represent 95% confidence intervals calculated using a Rao-Scott correction.
